# *AP1G2* Affects Mitotic Cycles of Female and Male Gametophytes in Arabidopsis

**DOI:** 10.3389/fpls.2022.924417

**Published:** 2022-07-07

**Authors:** Yongmei Zhou, Wenqin Fang, Ziqin Pang, Li-Yu Chen, Hanyang Cai, Noor-Ul- Ain, Men-Chi Chang, Ray Ming

**Affiliations:** ^1^FAFU and UIUC Joint Center for Genomics and Biotechnology, Key Laboratory of Sugarcane Biology and Genetic Breeding Ministry of Agriculture, Fujian Agriculture and Forestry University, Fuzhou, China; ^2^Fujian Provincial Key Laboratory of Haixia Applied Plant Systems Biology, Fujian Agriculture and Forestry University, Fuzhou, China; ^3^Department of Agronomy, National Taiwan University, Taipei, Taiwan; ^4^Department of Plant Biology, University of Illinois at Urbana-Champaign, Urbana, IL, United States

**Keywords:** *AP1G2*, Arabidopsis, Ca^2+^ signaling, gametogenesis, PICALM proteins

## Abstract

During sexual reproduction in flowering plants, haploid spores are formed from meiosis of spore mother cells. The spores then undergo mitosis, develop into female and male gametophytes, and give rise to seeds after fertilization. We identified a female sterile mutant *ap1g2-4* from EMS mutagenesis, and analyses of two T-DNA insertion mutants, *ap1g2-1*^+/−^ and *ap1g2-3*^−/−^, and detected a partial female and male sterility. The *ap1g2* mutant gametophyte development was arrested at one nuclear stage. A complementation test using a genomic sequence of *AP1G2* with its native promoter restored the function in the three *ap1g2* mutant lines. Transcriptome profiling of *ap1g2* ovules revealed that four genes encoding clathrin assembly proteins PICALM5A/B and PICALM9A/B, which were involved in endocytosis, were downregulated, which were confirmed to interact with AP1G2 through yeast two-hybrid assays and BIFC analysis. Our result also demonstrated that *RALFL4-8-15-19-26 CML16* and several calcium-dependent protein kinases, including *CPK14-16-17*, were all downregulated in the ovules of *ap1g2-1*^+/−^. Moreover, Ca^2+^ concentration was low in impaired gametophytes. Therefore, we proposed that through interaction with PICALM5A/B and PICALM9A/B, AP1G2 may mediate gametogenesis accompanied by Ca^2+^ signaling in Arabidopsis. Our findings revealed a crucial role of AP1G2 in female and male gametogenesis in Arabidopsis and enhanced our understanding of the molecular mechanisms underpinning sexual reproduction in flowering plants.

## Introduction

Plants have a life cycle variation between haploid and diploid phases, resulting in an alternation of generations. Angiosperms have evolved to produce a female gametophyte (FG; embryo sac) and a male gametophyte (MG; pollen). The female gametophyte plays an essential role in plant reproduction, including guiding pollen tube growth, fertilization, and seed development. In Arabidopsis, maize, and rice, genetic analyses have revealed that mutant defects were found at all stages of female gametophyte development, and analysis of the mutants began to reveal the characteristics of female gametophyte developmental programs (Yadegari and Drews, 2004; Drews and Koltunow, 2011; Bonnemaison et al., 2014). These studies improved our understanding of the regulatory network in the development of female gametophytes (Yadegari and Drews, 2004).

Large-scale screening for female sterile mutants has identified hundreds of female gametophyte mutants, while some mutants had defects in male gametophytes as well. Most of the characterized genes mediate essential functions (Yadegari and Drews, 2004; Pagnussat et al., 2005; Drews and Koltunow, 2011). Screening for female sterile mutants is a challenging task as it requires one additional generation to identify female sterile mutants. It also takes one additional generation to make crosses because such mutants can only maintain in heterozygous genotype. Our objective is to build a collection of female sterile mutants, ranging from the inception of carpel primordia, abortion of female reproduction organ, to pre- and post-meiosis mutations affection female gametophyte development to explore gene network controlling sex determination in male flowers.

The gene *AP1G2* was analyzed by whole-genome resequencing, and, apparently, it may encode a large subunit of adaptor protein complex-1 (AP-1). Adaptor protein (AP) complexes, the predominant coat proteins that link the membrane proteins with clathrin molecules that form the coat of a lipid vesicle, have been characterized in various eukaryotic cells. They interact with membrane proteins, such as different classes of cargo receptors in the process of generating a clathrin-coated vesicle (CCV). The structure of AP complexes is highly conserved across all eukaryotes and comprises two large subunits (α/γ/δ/ε and β), one medium μ subunit, and one small σ subunit, and the lack of any single APs subunit impairs the function of APs (Teh et al., 2013; Bonnemaison et al., 2015; Navarro Negredo et al., 2017). Among the APs, AP-1 plays a role in insoluble enzyme composition and some membrane proteins from trans-Golgi network (TGN) to endosomes and lysosomal transport (Bonnemaison et al., 2014; Wang et al., 2014, 2017). The γ subunit of AP-1 is encoded by *AP1G1* and *AP1G2* (Park et al., 2013). Earlier studies demonstrated that AP1G is crucial for synergid-controlled pollen tube reception and pollen development through mediating vacuolar remodeling (Feng et al., 2017; Wang et al., 2017). Here, we report new functions for *AP1G2* in gametogenesis. In this study, we characterized *AP1G2* in female and male gametogenesis. We identified three loci, At1g22410, At1g22730, and At1g23900 after mapping the causal mutation of a female sterile mutant obtained by EMS mutagenesis, and then screening and annotation of candidate SNPs showed that suppression of recombination occurred in all non-reference alleles (Ozias-Akins and Van Dijk, 2007). Confirmation of candidate genes was performed by studying single mutants of each candidate gene, and we found the T-DNA insertion lines of At1g23900 (*AP1G2*) showed mitosis malfunction during gametogenesis, which appeared in the sterile EMS mutant. We transformed the construct *ProAP1G2*:*AP1G2* into the EMS mutant, and the transgenic plants showed partial restoration infertility. Then, we did transcriptome profiling of *ap1g2* ovules, which revealed four downregulation of four genes encoding clathrin assembly proteins PICALM5A/B and PICALM9A/B, which have been reported to mediate endocytosis. Additional characterization by BIFC and yeast two-hybrid assays comfirmed their interaction *in vivo* and *in vitro*. Additionally, *RALFL* genes, *CML16*, and several calcium-dependent protein kinases, including *CPK14-16-17*, were all downregulated in the ovules of *ap1g2*. And Ca^2+^ concentration was low in impaired gametophytes. Therefore, we propose that, through interaction with PICALM5A/B and PICALM9A/B, AP1G2 mediates female and male gametogenesis by virtue of its regulation role during Ca^2+^ signaling in Arabidopsis.

## Materials and Methods

### Plant Materials and Growth Condition

The T-DNA insertion lines of *AP1G2*, SALK_032500 (*ap1g2-1*), SALK_137129 (*ap1g2-3*), and lines of At1g22410 and At1g22730 in Supplementary Table 2 were obtained from the Arabidopsis Biological Resource Center (ABRC). The pAKV:H2B-YFP marker line was kindly provided by W.C. Yang at Academician of the Chinese Academy of Sciences. All the seeds were sterilized with 75% ethanol, cold-treated at 4°C overnight, and germinated in Murashige and Skoog (MS) mediums. Seedlings were planted into the soil containing [(peat moss:perlite, 2:1 (v/v)] in plastic pots and put in an air-conditioned room under 22°C, 70% relative humidity, and a 16:8 (light:dark; L:D) photoperiod (Chen et al., 2017).

### Mutant Screening and Next-Generation Sequencing Analysis

The wild type and mutants used in the experiment were all from the Col-0 ecotype. Wild-type seeds were mutagenized with 40-m EMS for 8 h, and mutants induced by EMS were identified by screening plants from the second generation. The identified female-sterile mutants were backcrossed to the wild type to generate BC1 progeny and propagated by self-pollination to generate the BC1F2 segregating population.

Genomic DNA extracted from leaves of mutant and non-mutant plants was pooled. The libraries of both pools were constructed and sequenced by an Illumina HiSeq ™2500 platform at Novogene Corporation. The average sequencing depth was about 32-× coverage for both mutant and non-mutant pools. The reads we obtained from mutant and non-mutant pools were aligned with the Col-0 reference genome (*Arabidopsis thaliana*.TAIR10.21) by the software BWA, and SAMtools-mpileup was used to identify potential SNPs as described by Zhu et al. (2012). Single nucleotide polymorphisms (SNPs) of mutants were detected among BC1F2 segregants. Each candidate SNP was confirmed by PCR and sequencing of at least 12 mutants separately in BC2 progeny using primers as listed in Supplementary Table 1.

### Genotype Screening of T-DNA Insertion Line

Molecular identification of genotypes of the T-DNA insertion lines was performed as described by Pagnussat et al. (2007). The primers used for identification were designed using the online tool (http://signal.salk.edu/tdnaprimers.2.html). For all T-DNA insertion mutants, T-DNA-specific primer LBb1.3 (5′-ATTTTGCCGATTTCGGAAC-3′) was used, and the genomic sequence-specific primers were included in Supplementary Table 2. *INO* (*INNER NO OUTER*) is a key gene regulating the development of the outer integument (Meister et al., 2004; Sieber et al., 2004). To examine whether *INO* expression changed in EMS mutants, we collected the carpels from WT, wild-type plants from the BC1F2 population (*ap*1*g*2−4^+/+^ and *ap*1*g*2−4^+/−^), *ap*1*g*2−4^−/−^, *ap*1*g*2−4^−/−^/Pro:*AP1G*:*AP1G2, ap1g2-1*^+/−^, *ap1g2-3*^−/−^, *ap1g2-1*^+/−^*/*Pro:*AP1G*:*AP1G2*, and *ap1g2-3*^−/−^*/*Pro:*AP1G*:*AP1G2* to extract total RNA and perform qRT-PCR. Total RNA was extracted using Total RNA Kit I (OMEGA). The first-strand cDNA synthesis for real-time PCR and the latter were, respectively, performed using PrimeScript^®^ RT reagent Kit and SYBR Premix Ex Taq™ (Takara). The paired primers used for qRT-PCR were INO-F(5′ CAATGGTGGTGACTGTGAG 3′) and INO-R (5′ GCTTCTCAGGTGGTTTATT 3′) for *INO*, and Actin2F (5′TCCCTCAGCACATTCCAGCAGAT3′) and Actin2R (5′AACGATTCCTGGACCTGCCTCATC3′) for the reference gene *ACTIN2*. A *t*-test was used to analyze the differences between every two groups of samples. And qRT-PCR was performed for confirmation of mutation by T-DNA on target sites in T-DNA insertion lines using the primers AP1G2q-F (5′CTCCTGGCAAGCGGTAGT3′), AP1G2q-R (5′GCGAGGGAAGTTGCTGAC3′) for AP1G2 with Actin2F and Actin2R for the reference gene *ACTIN2*. The relative expression levels in different tissues were calculated with the cycle threshold methodology 2-ΔCt.

### Seed Set and Fertility Analysis

To analyze the seed set of the wild type and mutants, the total number of ovules and seeds was counted (*n* = 10), as described Alvarez and Smyth (1999). The seed set in each silique was the percentage of seeds to the total number of ovules. Statistical significance of the values was accessed using one-way ANOVA, followed by a least significant difference (LSD) test using a 95% confidence interval. For megagametophyte analysis, ovules were excised from different-sized pistils previously fixed in FAA (70% ethanol: acetic acid: 30% methylaldehyde, 9:0.5:0.5). They were cleared with chloral hydrate solution (chloral hydrate: glycerol: sterilized water, 8:1:2) and examined with the Olympus BX63 microscope equipped with DIC and phase-contrast optics.

### Vectors Construction and Plant Transformation

The genomic region-2kb before the start codon ATG corresponding to the putative AP1G2 promoter was amplified by PCR from wild-type genomic DNA using proAP1G2-F (5′-CACCAATACATGAGGGAAAGGTGAGA-3′) in combination with the reverse primer proAP1G2-R (5′-TTGGTCCACCGGCAACTTTA-3′). For the molecular complementation test, the 7,912 bp of the genomic fragment containing the promoter and the gene of *AP1G2* was amplified by PCR using the forward primer proAP1G2-F, in combination with the reverse primer AP1G2-R (5′-CAACCCGCGAGGGAAGTTG-3′) upstream of the stop codon. All PCR products were cloned in the pENTR/D/TOPO vector (Invitrogen). The generated entry vectors were subsequently used for generating the corresponding expression vectors PGWB533-GUS. Arabidopsis plants were transformed with agrobacterium tumefaciens strains GV3101 using the floral dip method (Clough and Bent, 1998). The presence of the transgene in T1 plants was confirmed by PCR using forward primer pro-F (5′AGTAGAGTAGGTAGCGTCAGAA-3′) for transgenic lines with promoter and gene-F (5′-ACGGAAAAGATGTATTAGAGG-3′) for complementation lines, and combined with the reverse primer GUS-R (5′-CGGCGAAATTCCATACCTG-3′).

### Cytological and Histochemical Analysis

For *ap1g2* phenotypic analysis, the whole inflorescences from wild type and *ap1g2* mutants were fixed in FAA fixative solution overnight, and transferred to sterilized water for 2 min. The ovaries were dissected on a slide and cleared in chloral hydrate solution and finally photographed using an Olympus BX63 microscope equipped with differential interference contrast (DIC) and phase-contrast optics. For β-Glucuronidase (GUS) staining, a GUS histochemical assays kit (Real-Time, China) was used following the manufacturer's protocol. Inflorescences and developing seedlings were incubated in a GUS staining buffer at 37°C overnight. Ovules were then washed three times in 75% ethanol for 7 h, followed by examination and photographs. To test the viability of the pollen, the anthers were collected at the anthesis stage and evaluated with the Alexander Red stain (Alexander, 1969). The development stage of pollen in wild type and mutants was analyzed for each subsequent bud in the same inflorescence from an open flower to the next 10 unopened buds. Anthers were placed on slides with DAPI staining solution ^(^0.02-M citric acid, 0.16-M Na_2_HPO_4_, 0.2 μgml^−1^ DAPI (D'ippolito et al., 2017), and photographed using an Olympus BX63 microscope.

### Confocal Microscopy of Ovules

To confirm that megagametogenesis was affected in *ap1g2* ovules, *ap1g2-1*^+/−^ and *ap1g2-3*^−/−^ were crossed with *pAKV*:*H2B-YFP* marker lines. We analyzed five progenies with an *ap1g2-1* and *ap1g2-3*, which showed partial sterility. The ovules at different flower stages were observed using a Leica TCS SP8X confocal microscope at an excitation wavelength of 488 nm. Emissions were collected at 500–530 nm to visualize yellow fluorescence protein (YFP). Anthers for each successive bud mounted into slides with DAPI staining solution were also observed using the confocal microscope at an excitation wavelength of 405 nm, and emissions were collected at 415–500 nm.

### Transcriptome Analysis

To obtain the expression profiles during female gametogenesis of the wild type and *ap1g2* mutants, we collected the ovules under a microscope from the wild type and the three *ap1g2* mutants at different stages for RNA-Seq. The RNA-seq data reads were mapped against the *Arabidopsis thaliana* reference genome by Hisat2 (Pertea et al., 2016). We used StringTie to calculate expression levels, which were normalized by fragment per kb of transcript per million fragments mapped (FPKM). Differentially expressed genes (DEGs) among samples were defined using fold change values by the expression of assembled transcripts. DEGs were selected on the criteria, having an adjusted *P* < 0.05 and fold change value > 2 by DESeq2.R (Anders and Huber, 2010). We visualized the ratios of each DEG of the three mutants by Ternary Plot Visualization (Bulgarelli et al., 2012; Pang et al., 2021). We used online software (https://www.omicshare.com/tools/Home/Soft/gogsea) to perform the GO enrichment analysis. We identified 20 gene clusters definedby Fuzzy c-means clustering using the expression profiles of the ovules from wild type and *ap1g2* mutant lines at the MMC, FG1, and FG3 stages (Gao et al., 2017).

DEGs in the top significant enriched GO terms that occurred in *ap1g2-1*^+/−^ were selected for STRING analysis (https://string-db.org). DEGs in the top significant enriched GO terms identified in *ap1g2-1*^+/−^ were selected for STRING analysis (https://string-db.org). The DEGs that occurred in Cluster 13 were also used for STRING analysis.

### Yeast Two-Hybrid Assay

Interaction between AP1G2 and the four PICALM proteins was tested in a yeast two-hybrid assay using a DUAL membrane pairwise interaction kit (DualsystemsBiotech). AP1G2 was cloned into the bait vector pBT3-N-AP1G2, and the four PICALM proteins were cloned into the prey vector pPR3-N (pPR3-N-PICALM5A, pPR3-N-PICALM5B, pPR3-N-PICALM9A, pPR3-N-PICALM9B). pBT3-N-AP1G2 and pOst1-NubI were co-transformed into the yeast strain NMY51 to test *AP1G2* expression. pBT3-N-AP1G2 and pPR3-N, pTSU2-APP and pPR3-N-PICALM5A, pTSU2-APP and pPR3-N-PICALM5B, pTSU2-APP and pPR3-N-PICALM9A, and pTSU2-APP and pPR3-N-PICALM9B were co-transformed into NMY51 as a negative control. pTSU2-APP and pNubG-Fe65 were co-transformed into NMY51 as a positive control. Interactions of pairs pBT3-N-AP1G2 and pPR3-N-PICALM5A, pBT3-N-AP1G2 and pPR3-N-PICALM5B, pBT3-N-AP1G2 and pPR3-N-PICALM9A, and pBT3-N-AP1G2 and pPR3-N-PICALM9B were assayed. All transformants were grown on synthetic dropout SD-Trp-Leu agar plates and SD-Trp-Leu-His-Ade agar plates for 4 days at 30°C.

### Bimolecular Fluorescence Complementation Analysis

The full-length AP1G2 and the four PICALM genes were amplified without stop codons, and cloned to YN and YC, respectively. Plasmids were transformed into *Agrobacterium* strain GV3101. Agrobacterium strains carrying the BiFC constructs were infiltrated into leaves of 5- to 6-week-old *Nicotianabenthamiana* plants. The YFP signals were examined by confocal microscopy (Leica TCS SP8X) in the leaf epidermis 24 h after the infiltration.

### Calcium Concentration Detection

A calcium ion detection kit was used to observe the changes in calcium concentration during female gametogenesis in the wild type and *ap*1*g*2−1^+/−^, which adopted the BBcellProbe F03 intracellular calcium ion concentration fluorescent probe to detect the change of calcium ion concentration. BBcellProbe F03 has very high cell permeability, and, after, it can combine with calcium ion in living cells and produce strong fluorescence. According to the instruction of this kit, we diluted the probe loading concentration by 1,000 times, using HBSS (Hank's Balanced Salt Solution; components: 140 mg/L CaCl_2_, 100 mg/L MgCl_2_.6H_2_O, 100 mg/L MgSO_4_.7H_2_O, 400 mg/L KCI, 60 mg/L KH_2_PO_4_, 350 mg/L NaHCO_3_, 8,000 mg/L NaCl, 48 mg/L Na2HPO, 1,000 mg/L D-Glucose). After washing the isolated ovules at different developing stages with a PBS buffer on slides, we added 25 ul F03 solution and incubated the slides at 22°C for 30 min. Approximately, a 25-ul PBS buffer was added instead of the F03 solution before incubation as negative control to determine the false positive staining reaction. The fluorescence signal of the ovules was detected using a Leica TCS SP8X confocal microscope at an excitation wavelength of 488 nm. Emissions were collected at 515–530 nm. To compare the Ca^2+^ level in wild-type and aborted embryo sacs, the software ImageJ (Schneider et al., 2012) was used to measure the mean fluorescent intensity.

The fresh inflorescences of the wild type and *ap1g2-1*^+/−^ were fixed in 2% glutaraldehyde (v/v) in a.1-M KH_2_PO_4_ buffer (pH 7.8), containing 1% potassium pyroantimonate (K_2_H_2_Sb_2_O_7_·4H_2_O) for overnight in 4°C (Qiu et al., 2005). The inflorescences were then washed three times with the same buffer containing 1% potassium pyroantimonate at 25°C and immersed for 4 h at 25°C in the same buffer, containing 1% (w/v) Osmium Tetraoxide. Inflorescences were then dehydrated using an acetone series [30, 50, 70, 85, 95, and 100% (v/v)]. Anthers and pistils at different developmental stages were finally embedded in Spurr's resin, and sections were observed with a transmission electron microscope (H-7650, HITACHI). We also fixed the wild-type inflorescences in the 2% glutaraldehyde, which was diluted with a.1-M KH_2_PO_4_ buffer (pH 7.8) and made sections as control. We examined the abundance of calcium precipitation in embryo sacs using imaging software (Simple PCI Version 6.6). Since calcium precipitates were not found in the central vacuoles, the central vacuoles were excluded when considering the areas of embryo sacs. So the abundance of calcium precipitates in wild-type embryo sacs only represented the average abundance in nuclear and cytoplasm. And, for the microspores, we only examined the abundance of young microspores with one nucleus centrally positioned, because, at this stage, the mutant microspores of *ap1g2-1*^+/−^ began to degenerate. The number of calcium precipitates per μm^2^ each of the two groups was analyzed using *t*-test.

## Results

### Mutant With Defects in Ovule Development

**Figure 1:**
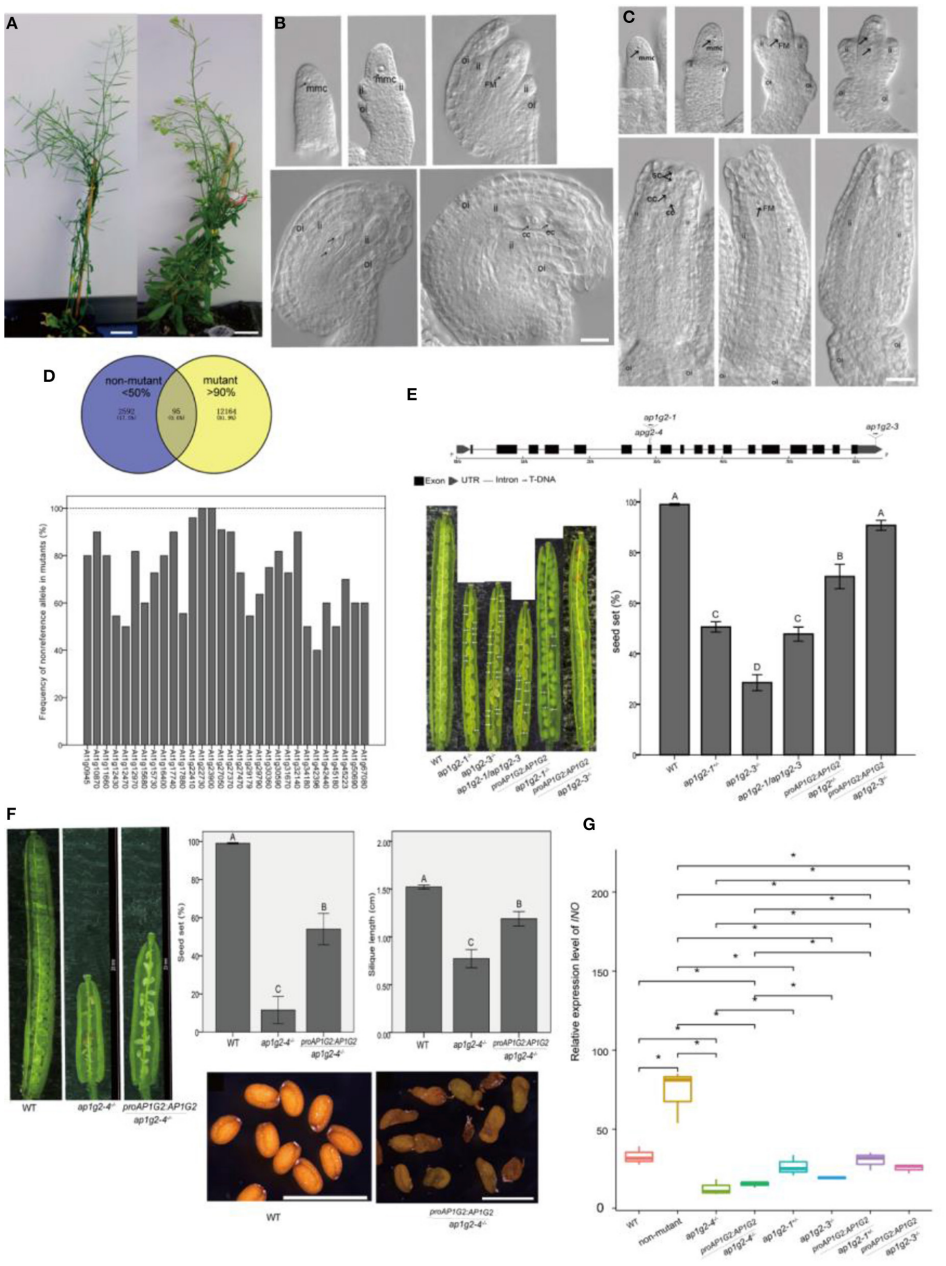
**(A–C)** Phenotypes of the EMS-induced sterile mutant. **(B,C)** Ovule development in WT and the EMS-induced sterile mutant. A scale bar = 20 mm in **(A)**. A scale bar = 20 μm in **(B,C)**, ii, inner integuments; oi, outer integuments; mmc, megaspore mother cell; FM, functional megaspore; cc, central cell; ec, egg cell; sc, synergid cells. **(D)** Ninety-five candidate SNPs with the non-reference allele >90% frequency in the mutant pool and <50% in the non-mutant pool, and frequency of the non-reference allele, which changed amino acid in homozygous mutants of BC2 progeny. **(E)** Schematic (Hu et al., 2015) representation of the gene *AP1G2*, with the positions of the various T-DNA insertions (*ap1g2-1* and *ap1g2-3*) and the EMS mutant harboring a G-to-A base change at nucleotide position 8444076 (*ap1g2-4*). The seed set of *ap1g2-1*^+/−^, *ap1g2-3*^−/−^, *apg2-1/apg2-3* mutants was significantly lower than that of wild-type plants and the genetic complementation lines of *ap1g2-1*^+/−^ and *ap1g2-3*^−/−^. **(F)** *AP1G2* driven by its native promoter could partially complement the infertility phenotype of *ap1g2-4*, but the seeds of transgenic plants had no heavy seed coat. Error bars indicate standard error. The capital letters above the bars mean very significant difference with the wild type (*p* < 0.01). **(G)** Quantitative qRT-PCR analysis of the *INO* expression. Each result was the average of three independent biological replicates. Error bars indicate standard error. *means significant difference (*p* < 0.05).

We obtained a sterile mutant from an ethyl methanesulfonate (EMS) mutagenesis screening, which showed shorter siliques with no seed set (Figure 1A). Cytological observations showed that the mutant had defects in the outer integument and embryo sac development (Figures 1B,C). About 56.3% of ovules were arrested at Stage FG1 (Christensen et al., 1998) in which the functional megaspore either persisted or degenerated after this stage. Approximately, 43.70% of ovules could undergo three mitoses and develop into mature embryo sac (Figure 1C).

### Mutation Identification

After backcross to wild-type plants, the self-pollinated BC1F2 plants were analyzed. The segregation ratio of wild type to mutants fits the expected 3:1 ratio (chi-square = 0.140, df = 1, *p* = 0.708). After backcross to the wild type, BC1 individuals were self-pollinated, and DNA of BC1F2 plants of mutants (*n* = 40) and non-mutants (*n* = 40) was pooled separately using the whole genome sequence as described by Nordstrom et al. (2013). We compared a causative mutation based on the frequency of the non-reference allele of a single nucleotide polymorphism (SNP) in the mutant and the non-mutant pools. If the non-reference allele of an SNP is the causal mutation, its frequency in the mutant pool should be 100% and about 33% in the non-mutant pool, and the SNPs associated with the causal gene should also display the high frequency of non-reference alleles in the mutant pool (Zhu et al., 2012). We selected 95 candidate SNPs (0.6% of total SNPs) with a frequency higher than 90% and lower than 50% in the mutant and non-mutant pools, respectively (Figure 1D). Among the 95 candidate SNPs, 81.05% of them were on Chromosome I, 0.07% on chromosome IV, 0.06% on Chromosome II, 0.02% on each chromosome III and chromosome V. To better understand the impact of SNPs on Chromosome I, we selected SNPs (30 associated genes) in coding regions that caused non-synonymous mutations or located in UTR (untranslated regions) for further analysis in the backcross BC2 progeny. As recombination events of the SNPs linked to the causal gene, each SNP was confirmed by PCR, and least 12 mutants were separately sequenced in the BC2 progeny. We found that At1g22730 and At1g23900 had 100% frequency of a non-reference allele in mutants, and At1g22410 had a frequency of 96%, making At1g22730, At1g23900, and At1g22410 candidate genes of the sterile mutant (Figure 1D).

### Candidate Genes Associated With Female Sterile Mutants

To determine which causal gene is associated with the sterile mutant, we studied several mutant lines with T-DNA insertion in each candidate gene. Among the T-DNA insertion lines, only two lines with insertions in *AP1G2* (At1g23900) showed the phenotype of a reduced seed set. *ap1g2-1* (SALK_032500) had T-DNA insertion in the 7th exon, and heterozygous plants *ap1g2-1*^+/−^ showed a 51.9% seed set (Figure 1E). For *ap1g2-3* (SALK_137129) with T-DNA insertion in 3′ UTR, 56 bp upstream from the poly A tail of the mRNA, we obtained homozygous plants (*ap1g2-3*^−/−^), with 23.27% of the seed set (Figure 1E). To understand whether there were other T-DNA insertions, genomic DNA of *ap1g2-1*^+/−^ was resequenced at a coverage rate of 24-× coverage of its whole genome, and we applied the software MaSuRCA 3.1.3 to assembly contigs (Zimin et al., 2013). Using the boundaries of the inserted fragment to align onto the assembled contigs, only one T-DNA insertion in the 7th exon of *AP1G2* was uncovered. Pollen grains from *ap1g2-3*^−/−^ were used as male parents to pollinate *ap1g2-1*^+/−^, by which heteroallelic homozygous mutants *ap1g2-1*^+/−^*/ap1g2-3*^+/−^were obtained with siliques containing 47.75% normal seeds (Figure 1E).

We carried out a complementation test using native promoter *ProAP1G2*, driving the wild-type allele. Five out of nineteen independent lines that were heterozygous for *ap1g2-1* and carried the transgene showed a significantly higher seed set. For *ap1g2-3*^−/−^, twenty-six independent lines were obtained, and eight lines complemented the *ap1g2-3*^−/−^ phenotype. The seed set of the *ap1g2-3*^−/−^mutant carrying the *ProAP1G2*:*AP1G2* constructwas −90.81%, very close to the vaule shown by the WT (99.04%) (Figure 1E). We also complemented the EMS mutant, which partially recovered its severely reduced fertility with an increase of 40% (Figure 1F). The ovules of the transgenic genotype still had no outer integuments, which led to the loss of episperm in seeds. Because *INO* (*INNER NO OUTER*) is a key gene regulating the development of the ovule outer integument and the defects of outer integument in *ap1g2-4*^−/−^ were very similar to those of *ino* mutants, so we doubted that the expression of this gene were affected in *ap1g2-4*^−/−^ (Meister et al., 2004; Sieber et al., 2004). The *INO* expression level was tested in the carpels from the wild-type plants, *ap1g2* mutants, and complementation lines, which showed *INO* was only regulated in *ap1g2-4*^−/−^ and *ap1g2-4*^−/−^/*ProAP1G2*:*AP1G2* (Figure 1G). These results revealed that the absence of the outer ovule integument was not caused by the mutation in AP1G2 of this EMS-induced mutant.

### Effect of *Ap1g2* on Female Gametophyte Development

**Figure 2:**
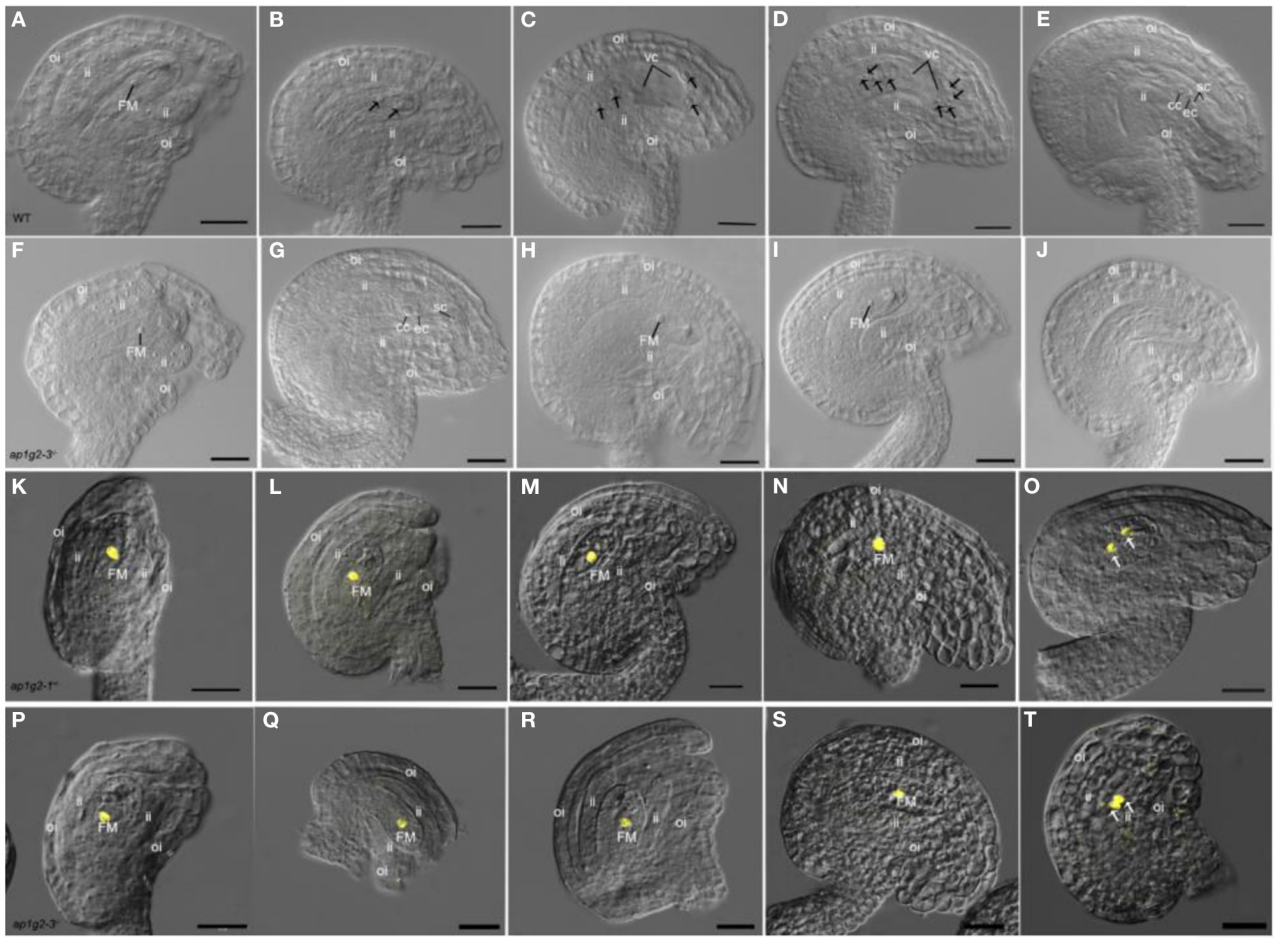
Female gametogenesis is impaired in ovules from *ap1g2-1*^+/−^ and *ap1g2-3*^−/−^ plants. **(A–E)** Ovule development in the wild-type, Stage FG1 **(A)**; Stage FG3 **(B)**; Stage FG4 **(C)**; Stage FG5 **(D)**; Stage FG7 **(E)**. **(F–J)** Ovules in *ap1g2-3*^−/−^, a normal ovule at Stage FG1 **(F)**; a mautrue ovule at FG7 aborted ovules arrested at FG1 **(H,I)**; **(J)** Aborted ovules without functional megaspore degenerated. **(K–O)** Analysis of *H2B-YFP* expression under the control of the *AKV* promoter in *ap1g2-1*^+/−^. **(L–N)** Aborted ovules arrested at FG1 in *ap1g2-1*^+/−^. **(O)** YFP signals in the wild-type ovule at the FG2 stage. **(P–T)** Analysis of *H2B-YFP* expression under the control of the *AKV* promoter in *ap1g2-3*^−/−^. **(Q–S)** Aborted ovules arrested at FG1 in *ap1g2-1*^+/−^. **(T)** The wild-type ovule at the FG2 stage. A scale bar = 20 μm, ii, inner integuments; oi, outer integuments; FM, functional megaspore; cc, central cell; ec, egg cell; sc, synergid cells.

To understand at which stage of the megagametophyte development might be affected in the *ap1g2* mutants, we observed the ovules from WT, *ap1g2-1*^+/−^, and *ap1g2-3*^−/−^ at different stages of development. The results showed that these plants were all able to produce functional megaspore cells (Figures 2A,F). Thereafter at the FG3 stage, wild-type plants contained a two-nucleate embryo sac, and continued to develop, producing four-nucleate embryo sacs and then mature embryo sacs (Figures 2B–E). About 50% of the ovules from *ap1g2-1*^+/−^ were arrested at Stage FG1, with the functional megaspore persisting or degenerating during development (Figures 2G–J). Ovules were observed (*n* = 989) in the *ap1g2-1*^+/−^ mutant, and the aborted ovules accounted for half of the total number (1:1, chi-square = 0.171, *p* = 0.679). While, in plants *ap1g2-3*^−/−^, most of the ovules contained a single cell in the nucellus or degenerated gradually (Figures 2G–J).

The *ANTI-KEVORKIAN* (*AKV*) cell-identity marker during megagametogenesis was used to confirm that the identity of the cell in the abortive ovules was the functional megaspore (Schmidt et al., 2011). The promoter *pAKV* is a gametophyte-specific promoter; therefore, the *pAKV*:*H2B-YFP* marker was specifically expressed in the nuclei of the functional megaspore and the developing gametophyte before cellularization (Su et al., 2017). We crossed *ap1g2-1*^+/−^ and *ap1g2-3*^−/−^ plants with *pAKV*:*H2B-YFP* marker lines and observed the ovules from the F1 plants with *ap1g2-1*^+/−^ allele and F2 plants with *ap1g2-3*^−/−^ allele. The YFP was expressed in the nuclei of the ovules at FG1 and wild-type ovules of *ap1g2-1*^+/−^ and *ap1g2-3*^−/−^,(Figures 2K,O,P,T) as well as the aborted ovules arrested at the mononucleate stage (Figures 2L–N,Q–S).

The female gametophyte development within a pistil is generally synchronous, with a relatively narrow range of variation in WT (Christensen et al., 1997; Shi et al., 2005). To investigate the developmental synchrony of female gametophytes in the pistils of *ap1g2* mutants, we emasculated flowers at the Stage 12 of development (Christensen et al., 1998), and, after 48–72 h, we fixed pistils from flowers of the wild type and mutants at different developmental stages. Compared with wild-type pistils, the developmental synchrony of female gametophytes in *ap1g2* was disturbed (Table 1, Supplementary Table 3). In *ap1g2-1*^+/−^ pistils, about half of the female gametophytes in each mutant pistil (P9–P14) either persisted at FG1 or degraded. While, in the *ap1g2-3*^−/−^, around 77.75% of all female gametophytes failed to undergo nuclear division (P9–P14).

**Table 1:** Synchrony of a female gametophyte in wild-type and *ap1g2-1*^+/−^ pistils.

**Pistil number**	**No. of FGs at developmental stages in wild-type pistils**									**No. of FGs at developmental stages in *ap1g2-1*^+/−^pistils**									
	**MMC**	**FG1**	**FG2**	**FG3**	**FG4**	**FG5**	**FG6**	**FG7**	**Total FGs**	**MMC**	**FG1**	**FG2**	**FG3**	**FG4**	**FG5**	**FG6**	**FG7**	**No nuclei**	**Total FGs**
P1	44								44	43									43
P2	33	12							45	21	28								49
P3	9	34							43	1	25	39							65
P4		36	2						38	1	25	3	9						38
P5		39	15	2	1				57		22	1	13	5		1		5	47
P6		25	10	4					39		22	1	6	12		1		5	47
P7		13	17	9	1				40		18	2	7	7	3			6	43
P8		9	6	26	10				51		17	2	3	3	3	4	4	6	42
P9				1	15	3	6	10	35		23		2	1		4	14	6	50
P10					11	10	11	10	42		18					3	18	9	48
P11							3	50	52		18					1	21	10	50
P12								51	51		17						20	19	56
P13								49	49		6						24	19	49
P14								50	50		5						21	20	46

### Effect of *Ap1g2* on Male Gametophyte Development

**Figure 3:**
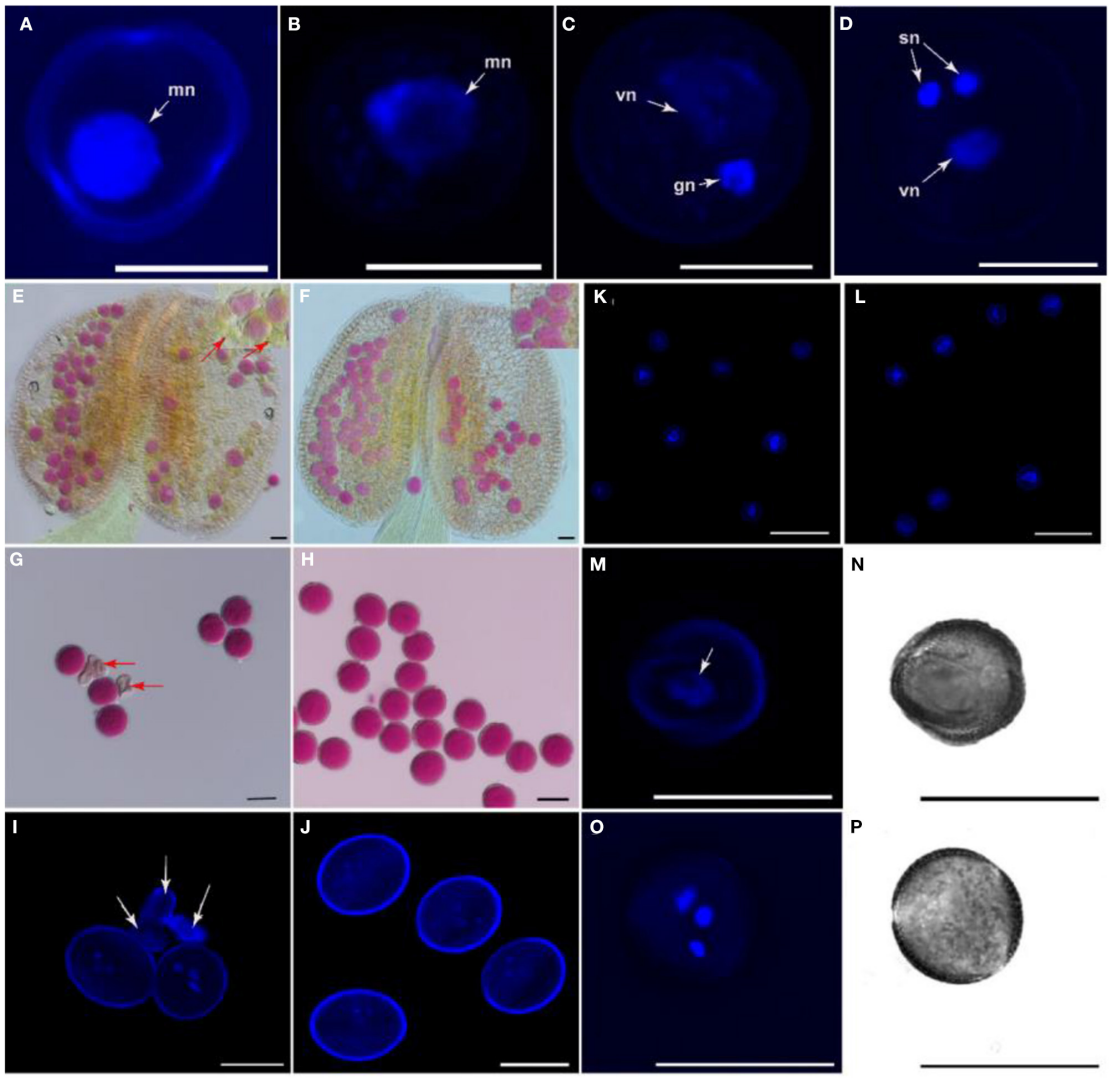
Morphology and viability of *apg2-1*^+/−^ mutant pollen grains. **(A–D)** The nuclear constitution of WT pollen grains through microgametogenesis developmental stages. Nuclei are indicated by arrows; mn, microspore nucleus; vn, vegetative nucleus; gn, generative nucleus; sn, sperm neclei. Bars = 10 μm. **(E,F)** Pollen viability by means of Alexander's staining in anthers from WT and *ap1g2-1*^+/−^, respectively. Arrows indicate impaired pollen grains. **(G,H)** Alexander's staining of mature pollen grains from WT and *ap1g2-1*^+/−^, respectively. Arrows indicate impaired pollen grains. **(I,J)** 40,6-Diamidino-2-phenylindole (DAPI) staining of pollen grains from buds at Stage 13 in WT and *ap1g2-1*^+/−^, respectively. Arrows indicate impaired pollen grains. **(K,L)** DAPI staining of microspores from buds at Stage 9 in WT and *ap1g2-1*^+/−^, respectively. **(M,O)** Nuclear constitution by means of DAPI staining in pollen grains in buds at Stage 12 from WT and *ap1g2-1*^+/−^, respectively. Arrows indicate nuclei. **(N,P)** Bright field images of **(M,O)**, respectively. Bars = 20 μm in **(E–P)**.

The development of the pollen grain begins with the expansion of the microspore (Figures 3A,B) and a large vacuole produced, companied by the migration of the microspore nuclear to a peripheral position against the cell wall. The microspore then undergoes a first asymmetric cell division (PMI)which results in a bicellular pollen gains (Figure 3C). Afterward, the smaller germ cell continues through a further round of mitosis (PMII), to produce twin sperm cells (Figure 3D). To analyze whether *ap1g2* led to pollen abortion, the viability of pollen grains was tested using Alexander Red stain. About 46.04% (*n* = 2,096) non-viable pollen was detected in mature anthers in *ap1g2-1*^+/−^ plants (Figures 3E–H), and 49.71% non-viable pollen was obtained for *ap1g2-3*^−/−^ (*n* = 1,750). However, the transgenic *ap1g2-1*^+/−^, carrying pro*AP1G2-AP1G2-GUS*, had a 71.22% (*n* = 300) viability rate, and the transgenic *ap1g2-3*^−/−^ plants had a similar-level viability rate as the wild type (98.70%, *n* = 230), resulting in 1.64% (*n* = 600) aborted pollen.

We used 4′,6-diamidino-2-phenylindole (DAPI) staining to trace the pollen development in wild-type plants and *ap1g2-1*^+/−^, which was a single T-DNA insertion mutant. The normal mature pollen grains from wild type and *ap1g2-1*^+/−^ contained a vegetative nucleus and two generative nuclei (Figures 3I,J), while about half of the pollen grains from *ap1g2-1*^+/−^ did not possess a nuclear fluorescence signal as the pollen was abnormal and shriveled (Figure 3J). Although at the microspore stage, pollen grains in both WT and *ap1g2-1*^+/−^ showed normal single nucleus fluorescence (Figures 3K,L), nearly half of the microspores of *ap1g2-1*^+/−^did not undergo nuclear polarization before pollen mitosis but still showed unicellular and shriveled microspores (Figures 3O,P) when tricellular pollen grains had formed in the wild type (Figures 3M,N).

About half the pollen grains from *ap1g2-3*^−/−^ were shown to be wrinkled shaped under scanning electron microscopy (SEM) (Supplementary Figures 2B,D), in contrast to those of wild type (Supplementary Figures 1A,C). Solid pollen germination mediums (Rodriguez Enriquez et al., 2013) were used to culture the pollen grains from WT and *ap1g2-3*^−/−^, and we obtained 72.22% (*n* = 180) and 30% (*n* = 180) germination, respectively (Supplementary Figures 1E,F).

### *AP1G2* Expression Pattern

**Figure 4:**
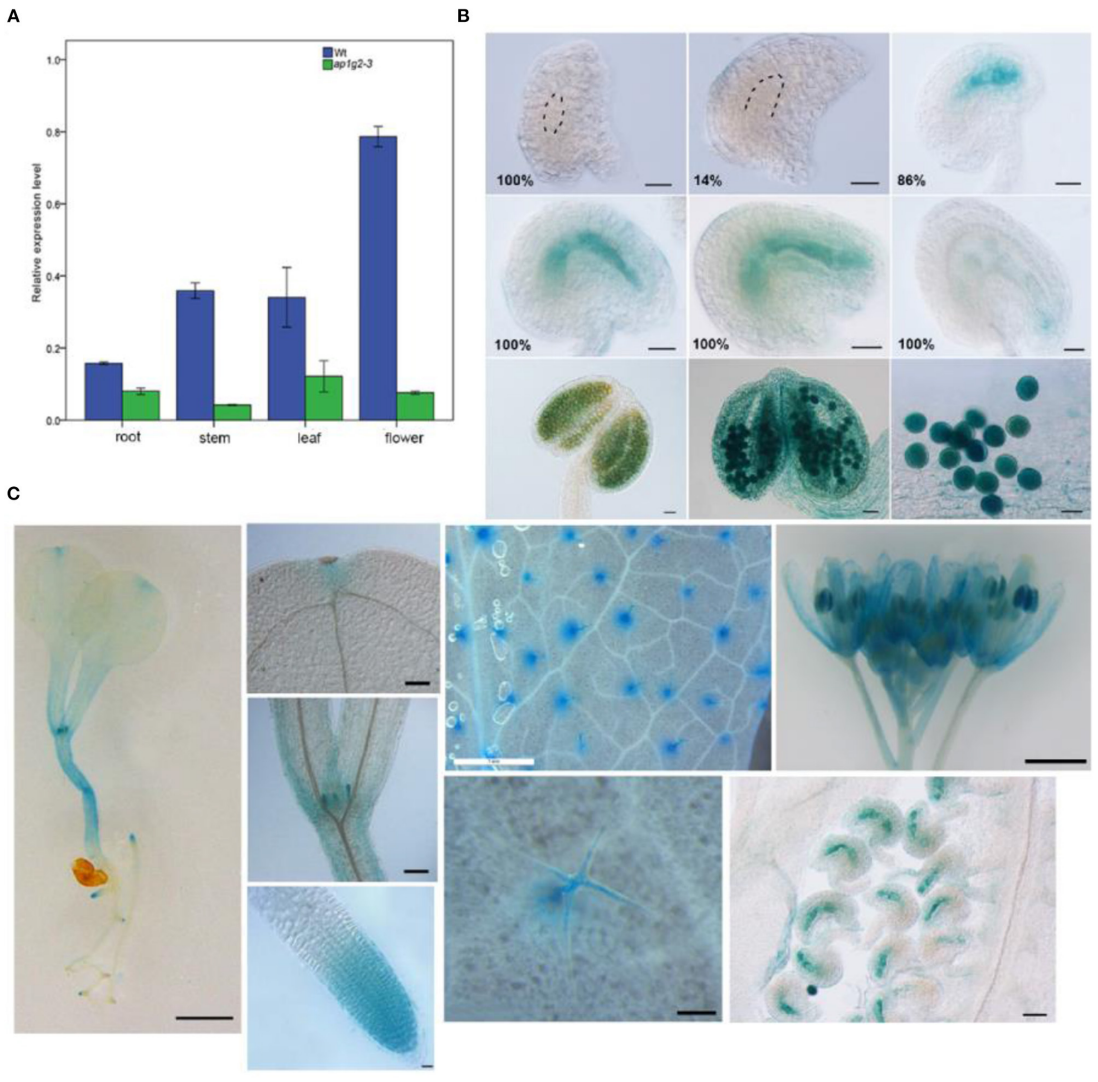
**(A)** Quantitative qRT-PCR analysis of the *AP1G2* gene expression. Each result was the average of three independent biological replicates. Error bars indicate standard error. **(B)** Activity of *AP1G2* in ovules and anthers. One hundred percent of ovules containing female gamete at Stage FG1 and 14% of ovules containing a female gametophyte at FG3 were not detected *GUS* expression. One hundred percent ovules expressed GUS strongly at FG4 to FG7 until the process of embryogenesis. GUS staining in anthers and pollen grains. Bars = 20 μm. **(C)** Pro*AP1G2:GUS* expression in the seedlings, leaves, and flowers. Bar = 1 cm in the pictures of 8-day-old seeding, leaves, and inflorescence. Bar = 20 μm in in the pictures of leaf primordial, apical meristem, trichomes, and ovules.

The qRT-PCR analysis revealed that *AP1G2* expression was found in a variety of tissues, including roots, leaves, stems, and flowers, where flowers revealed the highest relative expression, followed by stems and leaves (Figure 4A). For *ap1g2-3*^−/−^ mutants with the T-DNA insertion in 3′ UTR, the expression levels were significantly downregulated as compared with the wild type using *t*-test (*p* < 0.01).

Spatial and temporal GUS expression profiles of transgenic *AP1G2* plants were observed. Twenty-three independent lines of the T2 generation were analyzed, of which 5 showed GUS expression in the female gametophyte, and GUS expression was detected after the big vacuole formed and remained until embryogenesis began; in later stages, GUS expression was reduced (Figure 4B), while the same pattern was observed in anthers. Pro*AP1G2*:*GUS* was specifically expressed in the male gametophyte at maturation in all independent lines (Figure 4B). Additionally, Pro*AP1G2*:*GUS* was expressed at the 8–10-day seedling stage, and expression was also detected in hypocotyle, leaves, leaf primordia, shoot apical meristem, flowers, anthers, filament, and pedicles. GUS expression was also observed in root tips, while strong GUS staining was noted in trichomes (Figure 4C).

### Transcriptomic Profiling of *Ap1g2* Ovules

To recover the mechanism that how *AP1G2* mediated the process of female gametogenesis, we collected the ovules from the wild type and the three *ap1g2* mutants at different stages for RNA-Seq analysis. Expression profiles were used for principal component analysis (PCA), which revealed that *ap1g2-3*^−/−^ expressed at MMC and FG3 stages, which were significantly different from that of the wild type (Supplementary Figure 2A). For differentially expressed genes (DEGs), the number of upregulated DEGs of *ap1g2-1*^+/−^ and *ap1g2-1*^+/−^*/ap1g2-3*^+/−^ peaked at MMC stages and decreased significantly at FG1 and FG3 stages when the downregulated DEGs increased. The number of downregulated DEGs of *ap1g2-3*^−/−^ at FG1 and FG7 stages also increased compared with the MMC stage (Supplementary Figure 2B). The total number of the DEGs at each stage of *ap1g2-1*^+/−^, *ap1g2-1*^+/−^*/ap1g2-3*^+/−^, and *ap1g2-3*^−/−^ was 648, 636, and 837, respectively. Among them, there were 164 common DEGs at all phases of the three mutant lines (Supplementary Figure 2C).

**Figure 5:**
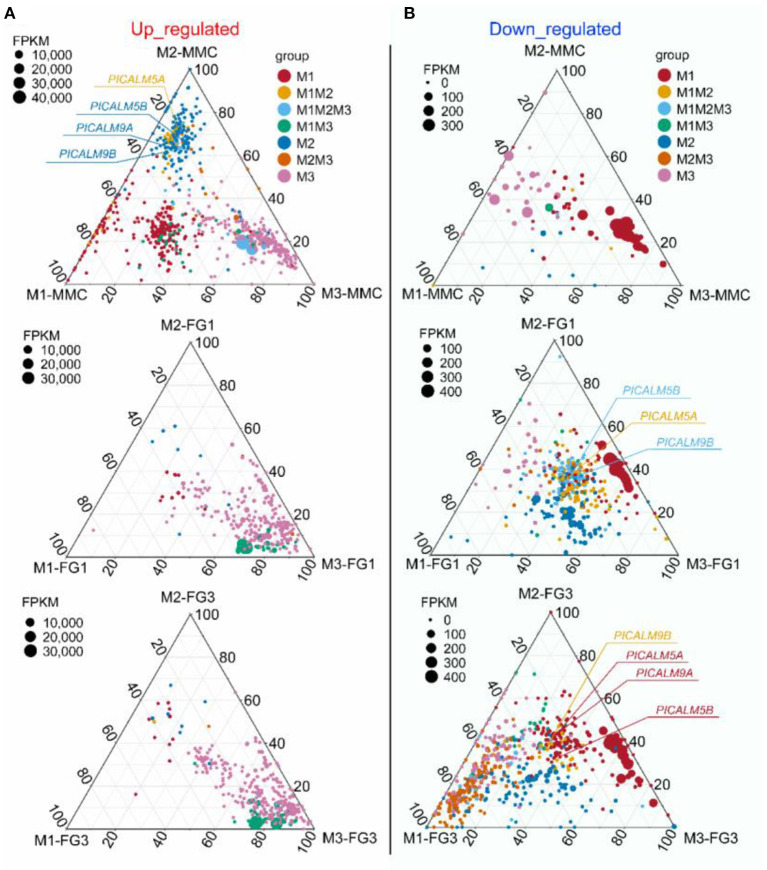
A ternary plot of all DEGs from *ap1g2-1*^+/−^ (M1), *ap1g2-1/ap1g2-3* (M2), and *ap1g2-3*^−/−^(M3) at MMC, FG1, and FG3 stages. **(A)** The upregulated DEGs of the three *ap1g2* mutants at each stage; **(B)** The downregulated DEGs of the three *ap1g2* mutants at each stage. Each circle represented one DEG. The size of each circle represented one DEG's FPKM. The position of each circle was determined by the contribution of the indicated compartments to the total FPKM. The dotted grid and numbers inside the plot indicated 20% increments of contribution from each gene. Red circles denoted the specific DEGs of *ap1g2-1*^+/−^. Yellow circles denoted the overlapping DEGs of *ap1g2-1*^+/−^ and *ap1g2-1*^+/−^*/ap1g2-3*^+/−^. Light blue circles denoted the overlapping DEGs of *ap1g2-1*^+/−^ and *ap1g2-1*^+/−^*/ap1g2-3*^+/−^ and *ap1g2-3*^−/−^. Green circles represented the overlapping DEGs of *ap1g2-1*^+/−^ and *ap1g2-3*^−/−^. Dark blue circles represented the specific DEGs of *ap1g2-1*^+/−^*/ap1g2-3*^+/−^. Orange circles represented the overlapping DEGs of *ap1g2-1*^+/−^*/ap1g2-3*^+/−^ and *ap1g2-3*^−/−^, and pink circles represented the specific DEGs of *ap1g2-3*^−/−^.

To find out whether the DEGs identified belong to a particular mutant or are common in two or three mutants, all DEGs of the three mutant lines at each stage were divided into seven groups (**Figure 10**). For example, *PHOSPHATIDYLINOSITOL BINDING CLATHRIN ASSEMBLY PROTEIN 5A* (*PICALM5A*) was significantly upregulated in both *ap1g2-1*^+/−^ and *ap1g2-1*^+/−^*/ap1g2-3*^+/−^ at the MMC stage, and *PICALM5B* was upregulated in all *ap1g2-1* at the MMC stage (Figure 5A).

**Figure 6:**
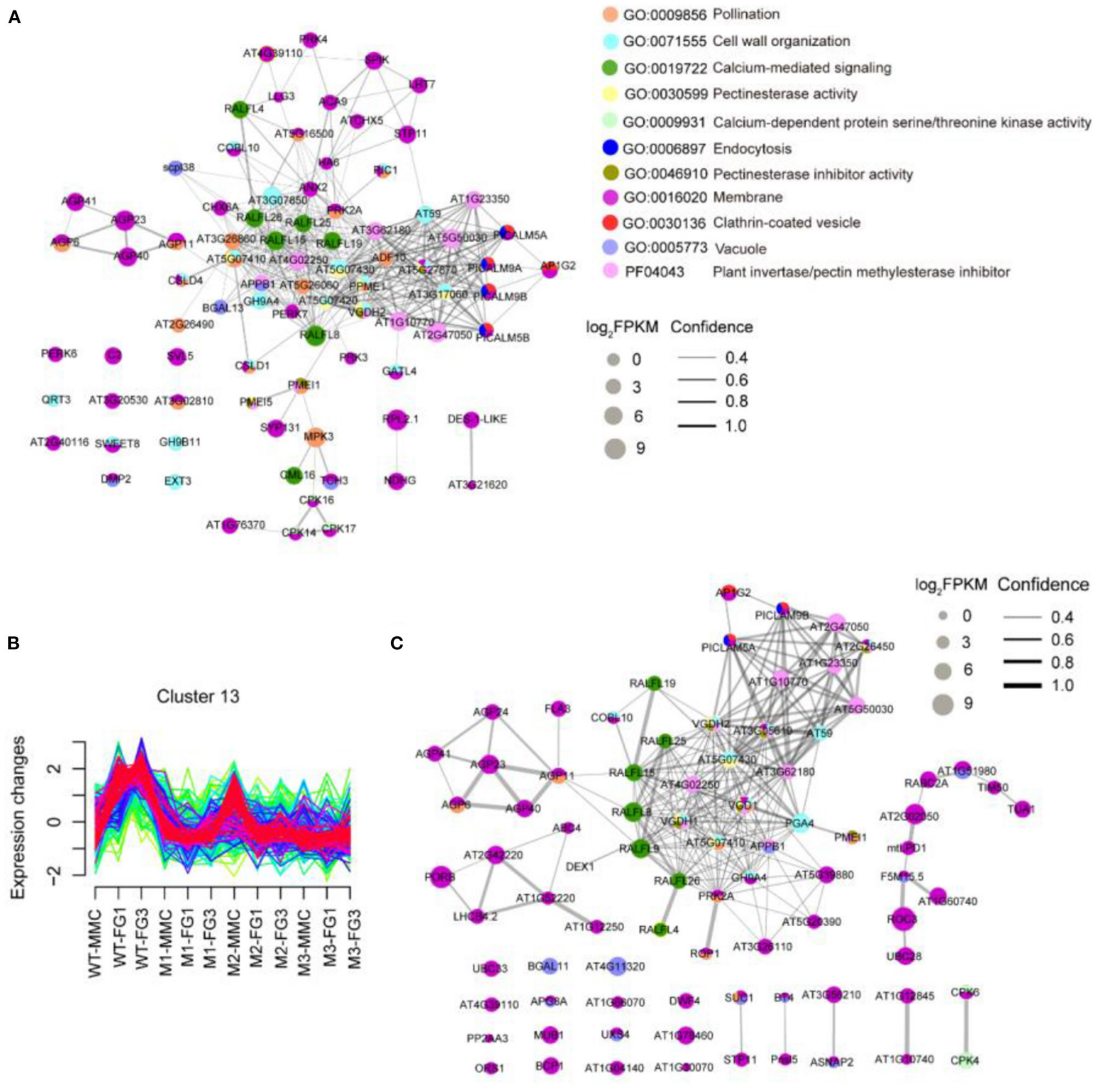
STRING protein-protein interaction network. **(A)** The STRING analysis downregulated DEGs of *ap1g2-1*^+/−^. **(B)** Cluster 13 showed an increase of the expression level at FG1 in wild-type ovules but repressed in the *ap1g2* mutants at various stages. The y axis represented the standardized FPKM value of genes, and the x axis represented the different samples. **(C)** Genes of Cluster 13 that were enriched on the above GO terms in **(A)** were used for STRING analysis. Colored nodes from **(A,C)** indicated the proteins enriched on different GO terms. The size of each node indicated the normalized FPKM value. Lines between nodes represented both functional and physical protein associations. The line thickness indicated the confidence of association.

From the MMC to the FG3 stage, there were 40, 66, and 21 common DEGs among the three mutant lines, respectively. At the FG1 stage, 66 DEGs were all downregulated in three mutant lines (Figure 5B). GO enrichment analysis revealed that downregulated DEGs of *ap1g2-1*^+/−^ were enriched on pollination, cell wall organization, calcium-mediated signaling, and pectinesterase activity, calcium-dependent protein serine/threonine kinase activity, endocytosis, pectinesterase inhibitor activity, membrane, clathrin-coated vesicles, and vacuoles. These proteins, together with the proteins containing the domain of invertase/pectin methylesterase inhibitor, were used for STRING analysis (Figure 6A). Among the DEGs, *PICLAM9B*, and *PICLAM5B* were upregulated at the MMC stage but downregulated at the following stage when the development of partial ovules was arrested. The results showed that AP1G2, PICALM5A/B, and PICALM9A/B were located on the membrane and clathin-coated vesicles. And the four PICALM proteins, which have been reported to be involved in endocytosis, were predicted to interact with AP1G2 because the γ-adaptins and PICALM6 were found interacting in *Mus*musculus (Wang et al., 1995).

We also defined 20 gene clusters by Fuzzy c-means clustering (Supplementary Figure 3) using the expression profiles to show the expression trends of different genes at developing stages, in which Cluster 13 containing 270 genes showed decreased expression profiles of *ap1g2* mutants at MMC and FG1 stages (Figure 6B). Eighty-three genes from Cluster 13 that were enriched on the above-mentioned GO terms or contained the domain of the invertase/pectin methylesterase inhibitor were selected for a STRING protein network map (Figure 6C). AP1G2 showed direct interactions with PICALM5A and PICALM9B (located in the periphery of the network diagram). Whereas, the phosphatidylinositol binding clathrin assembly proteins were predicted to have associations with those proteins that were putative pectin methylesterase/invertase inhibitors like AT2G47050, AT1G23350, AT1G10770, or other proteins involved in pectinesterase activity and cell wall organization, including AT5G07430 and *VANGUARD 1 HOMOLOG 2* (VGDH2) (Figures 6A,C).

Some RALFL (Rapid Alkalinizationfactor-like) peptides, which induce a rapid increase in cytoplasmic calcium (Haruta et al., 2008; Somoza, 2021), also occurred in both network maps *Calmodulin Like 16* (*CML16*), and calcium-dependent protein kinase genes, including *CPK14, CPK16*, and *CPK17*, which are calcium-binding genes, were downregulated in the *ap1g2-1*^+/−^ mutant. In both network diagrams, members of the AGP (arabinogalactan protein) family appeared to be in connection with *RALFL* peptides (Figure 6).

### *AP1G2* Interacted With *PICALM5A/B* and *PICALM9A/B*

**Figure 7:**
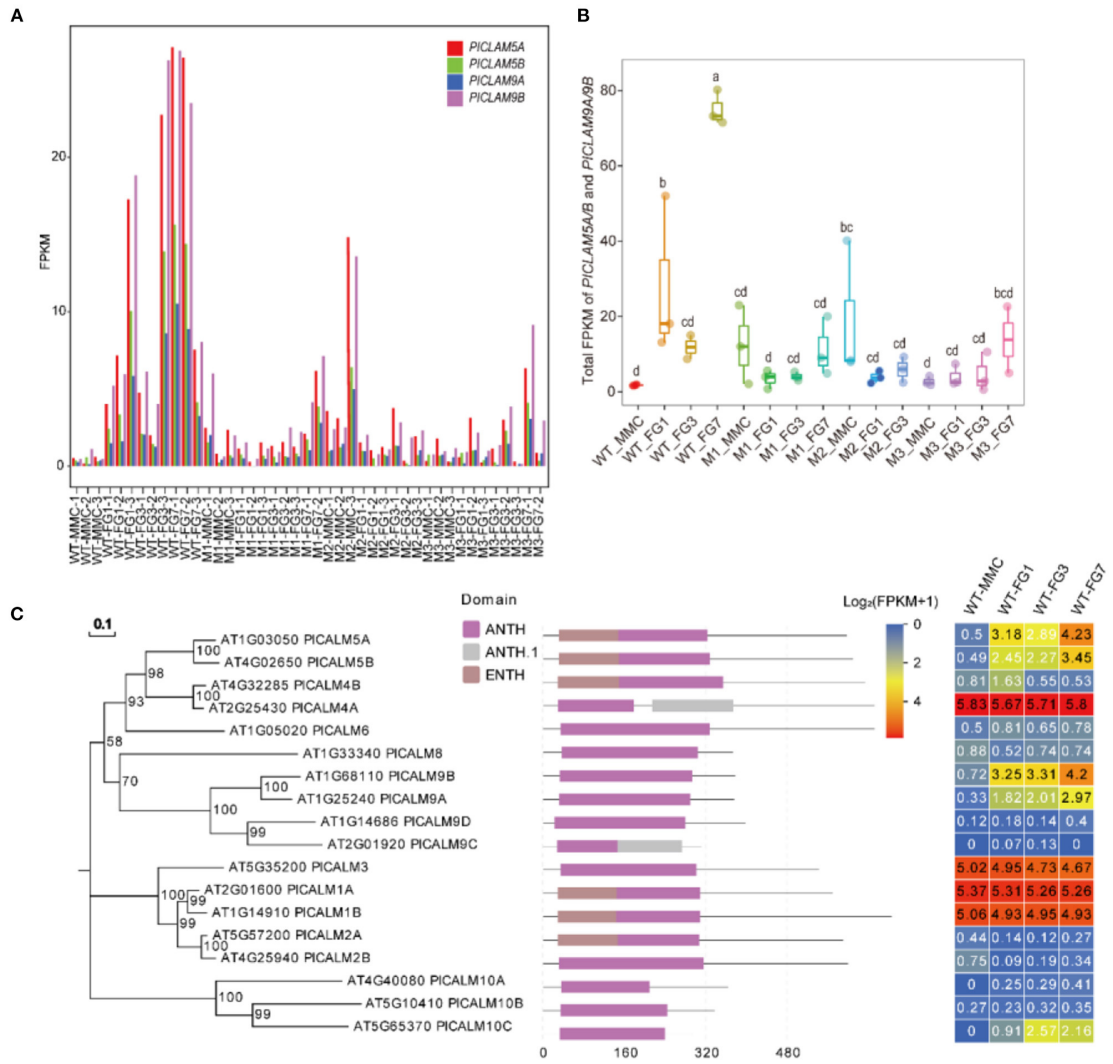
**(A)** FPKM value of *PICALM5A/B* and *PICALM9A*/*B* at each stage in WT, M1, M2, and M3. **(B)** The total FPKM values of the four PICALMs at each stage in WT, M1, M2, and M3. The lowercase letters above the bars mean significant difference (*p* < 0.05). **(C)** The expression profiles of each member of the ANTH family. Bootstraps (based on 1,000 replications) of the un-rooted phylogenetic tree are indicated at each node (bar = 0.1 amino acid substitutions per site).

*PICALM5A*/*B* and *PICALM9A/B* belong to the ANTH family. In the wild type, the four ANTH genes were barely expressed at the MMC stage in the wild type, but their expression levels dramatically increased at the FG1 stage (Figure 7), while *PICALM5A* and *PICALM9A/B* were upregulated at the MMC stage of *ap1g2* mutants. *PICALM5B* and *PICALM9B* were downregulated in all mutants at Stage FG1, and *PICALM5A* was also downregulated in *ap1g2-1*^+/−^ (Figures 6, 7A). At the FG7 stage of *ap1g2-1*^+/−^, the four genes showed lower expression than those of the wild type. Given the four genes may act redundantly, the total expression level of *PICALM5A*/*B* and *PICALM9A/B* at each stage of wild type; *ap1g2* mutants were compared, which showed that the total expression level was significantly reduced at FG1 and FG7 stages in *ap1g2* mutants. And qRT-PCR analysis also revealed that *PICALM5A/B* and *PICALM9A/B* were downregulated at developing ovules in *ap1g2* (Supplementary Figure 4). In the ANTH family, only *PICALM4A* and *PICALM9C* have two ANTH domains, while the other members contained one. Among the ANTH family members, *PICALM5A/B, PICALM1A/B*, and *PICALM2A* have an ENTH domain, which is also a part of their ANTH domains. The genes of the ANTH family showed three expression patterns during the development of ovules in the wild type. *PICALM4A, PICALM3*, and *PICALM1A*/*B* maintained consistent expressions that might play very basic functions. Whereas members, including *PICALM5A/B, PICALM9A/B*, and *PICALM10C*, seemed to be involved in the megagametogenesis and pollination, since they showed low expressions before the FG1 stage, but kept relatively higher expression levels during the mitosis process of the female gametophyte and peaked at FG7 when the female gametophytes matured and were ready for double fertilization (Figure 7C). While *PICALM4B, PICALM6, PICALM9C/D, PICALM2A/B*, and *PICALM10A/B* showed extremely low transcriptional activity throughout the ovule development.

**Figure 8:**
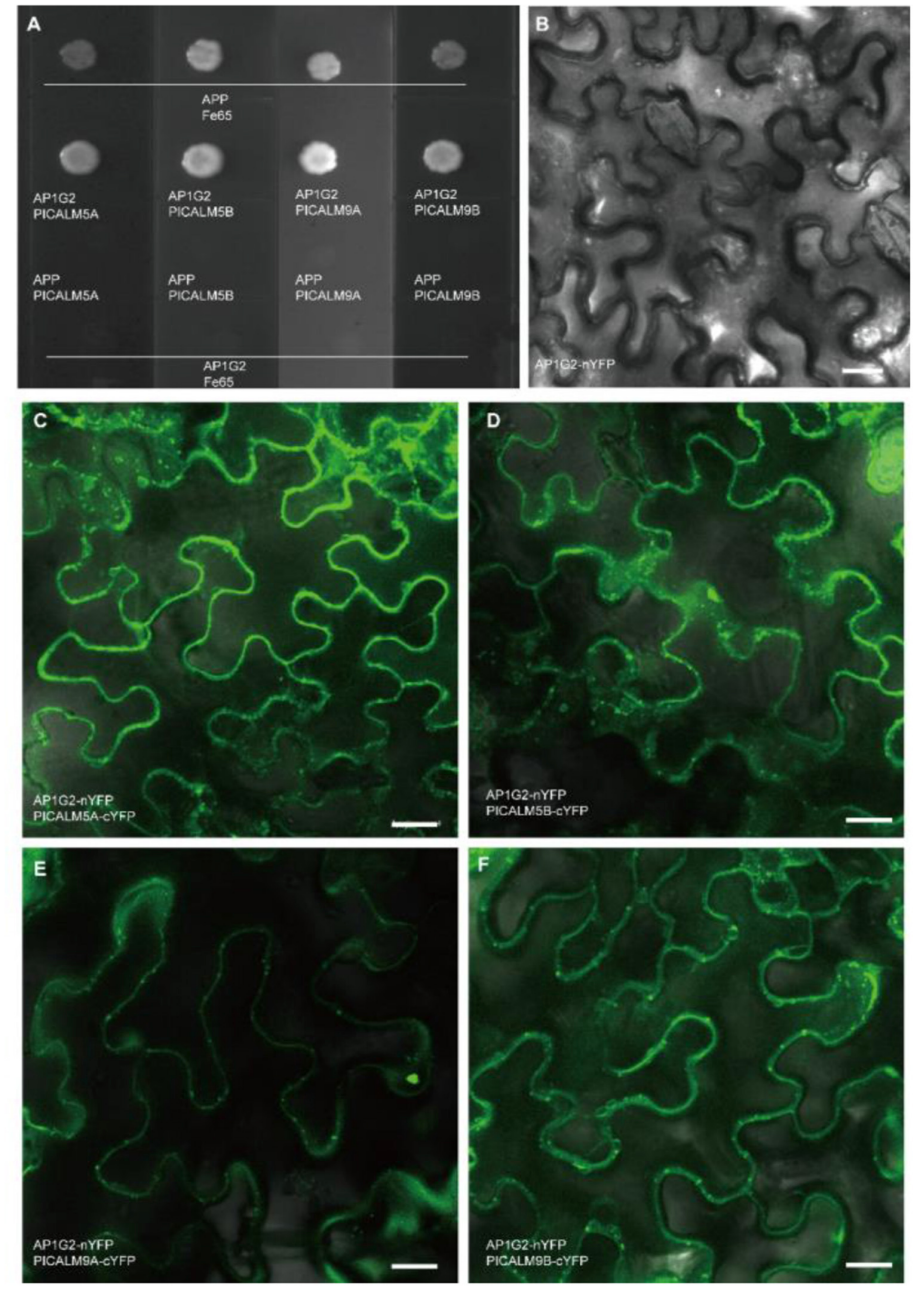
**(A)** The protein-protein interactions of AP1G2 and PICALM5A/B and PICALM9A/B in yeast. Vectors pTSU2-APP and pNubG-Fe65 were positive control bait and prey, respectively. **(B–F)** BiFC analysis of AP1G2 and PICALM6A/B and PICALM9A/B in tobacco leaf epidermis. **(B)** is a negative control. Confocal images showing YFP fluorescence indicated interaction.

To confirm the interactions between AP1G2 and the four candidate targets, we used split-ubiquitin yeast two-hybrid assays and a Bimolecular Fluorescence Complementation (BiFC) assay, which turned out that *AP1G2* interacted with PICALM5A/B and PICALM9A/B in yeast and tobacco (Figures 8A–F). In tobacco leaf epidermal cells, we found the AP1G2, PICALM5A/B, and PICALM9A/B mainly localized and interact at the cellular plasma membrane and vesicles close to the plasma membrane (Figures 8C–F).

### Calcium Dynamics in the Developing Embryo Sacs and Pollen Grains in *Ap1g2-1^+/−^*

**Figure 9:**
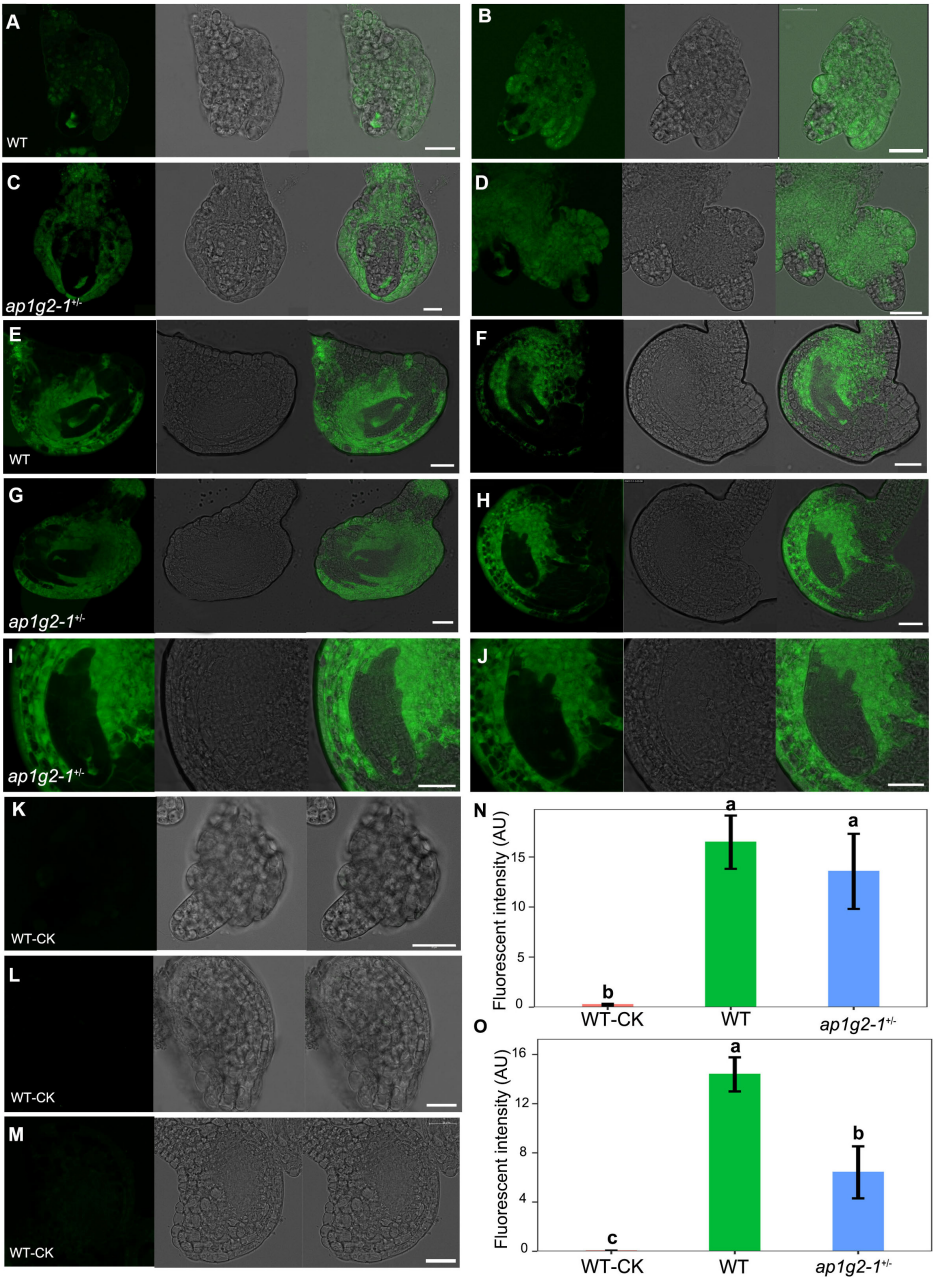
Calcium dynamics during female gametogenesis in WT and *ap1g2-1*^+/−^. **(A,B)** and **(C,D)** ovules at FG1 from WT and *ap1g2-1*^+/−^ dyed by an intracellular calcium ion concentration fluorescent probe. **(E,F)** ovules around at FG3 and FG4 from WT, respectively. **(G,H)** Aborted embryo sacs from *ap1g2-1*^+/−^. **(I,J)** were magnification of **(E,F)**. **(K–M)** were the ovules from the wild type without dyeing. **(N)** Quantification of the Ca2^+^ level in ovules of WT and *ap1g2-1*^+/−^ at the FG1 stage. **(O)** Quantification of the Ca2^+^ level in ovules at later stages from WT and aborted ovules of *ap1g2-1*^+/−^. Bar = 20 um.

The expression profiles of RNA-Seq data showed that calcium-dependent protein kinases, i.e., *CPK14, CPK17*, and *CPK36*, were downregulated in *ap1g2* mutants, and several RALFL peptides were also downregulated and grouped in Cluster 13 (Figures 6A,C); we examined calcium concentrations in the ovules and microspores of the wild type and *ap*1*g*2−1^+/−^. We used a calcium ion detection kit to observe the calcium dynamic changes during female gametogenesis in the wild type and *ap*1*g*2−1^+/−^, which adopted a fluorescent probe to detect the change of calcium ion concentration. The fluorescence signal was detected at the degenerated micropylar-most megaspores of ovules in both wild type (Figures 9A,B) and *ap1g2-1*^+/−^ (Figures 9C,D). At later development stages, the wild-type embryo sacs showed a higher concentration of calcium ion than that of the aborted embryo sacs of *ap1g2-1*^+/−^ (Figures 9E–O).

**Figure 10:**
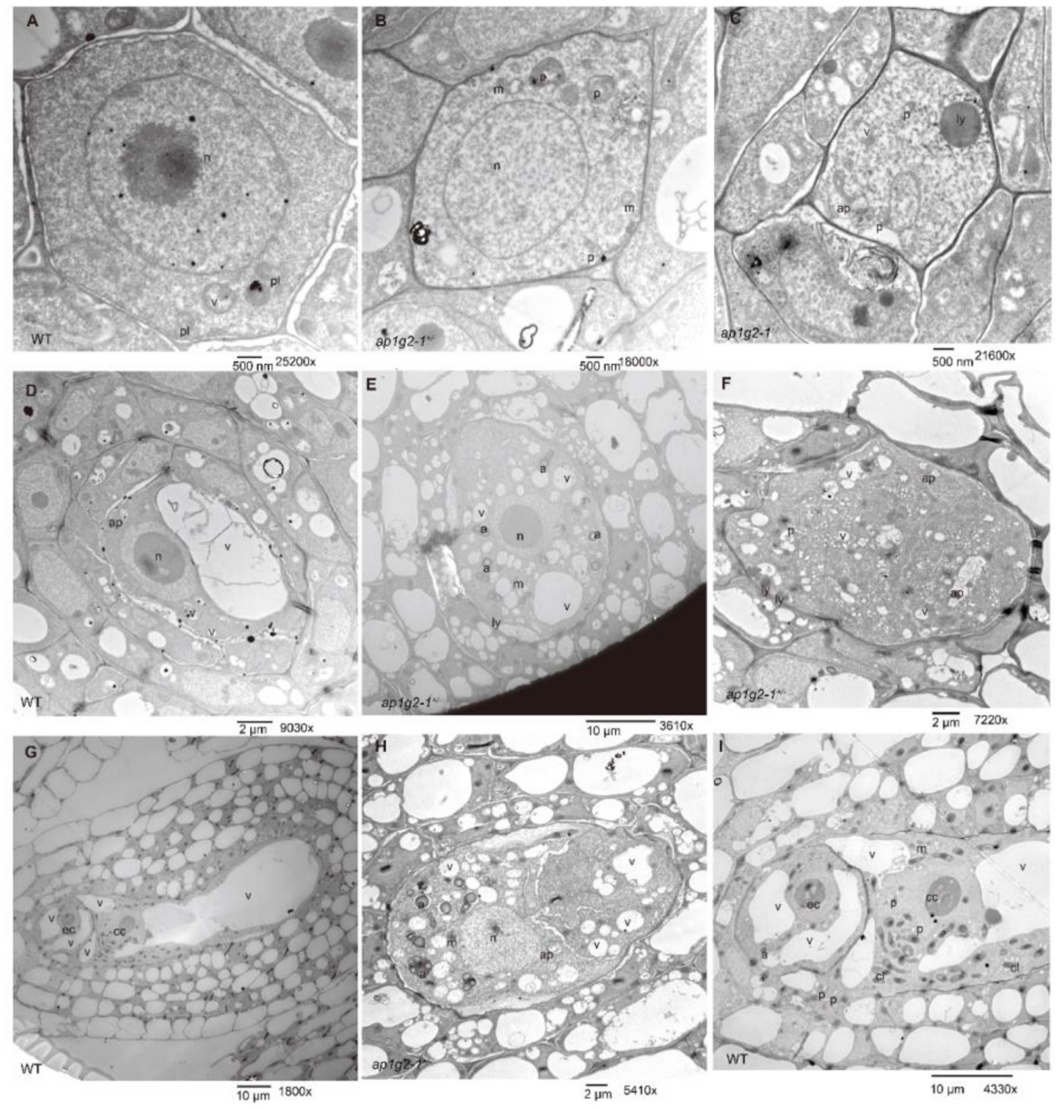
TEM micrographs of the pyroantimonate-labeled ovules from the wild type and *ap1g2-1*^+/−^. **(A,D,G)** Showed the ovules of the wild type at MMC, FG3, and FG7, respectively. **(B,C)** showed the ovules at the MMC stage from *ap1g2-1*^+/−^. **(E,F,H)** showed the aborted ovules from *ap1g2-1*^+/−^, while **(I)** was a magnification of **(G)**. n, nucleus; p, proplastids; v,vacuoles; m, mitochondria; ap, autophagosomes; a, amyloplasts; ly, lysosomes; cl, chloroplasts; ec, egg cell; cc, central cell. The black dots were calcium precipitates.

We also treated pistils and anthers at different stages from the wild type and *ap1g2-1*^+/−^ with potassium pyroantimonate to localize loosely bound calcium, and observed the calcium precipitate through a transmission electron microscope (TEM). In the wild-type embryo sacs, there were many calcium precipitates that were distributed (Figures 10A,D,G,I, Supplementary Figure 4A), whereas *ap1g2-1*^+/−^ abnormal embryo sacs contained fewer and smaller calcium precipitates (Figures 10C,E,F,H, Supplementary Figure 4A).

Additionally, we found the aborted embryo sacs at FG1 that could not form a large central vacuole, which polarizes the two daughter nuclei in a wild-type embryo sac with a lot of randomly distributed small vacuoles. There were large amounts of amyloplasts that appeared in the abortive embryo sacs but very few in the wild-type ones (Figure 10).

**Figure 11:**
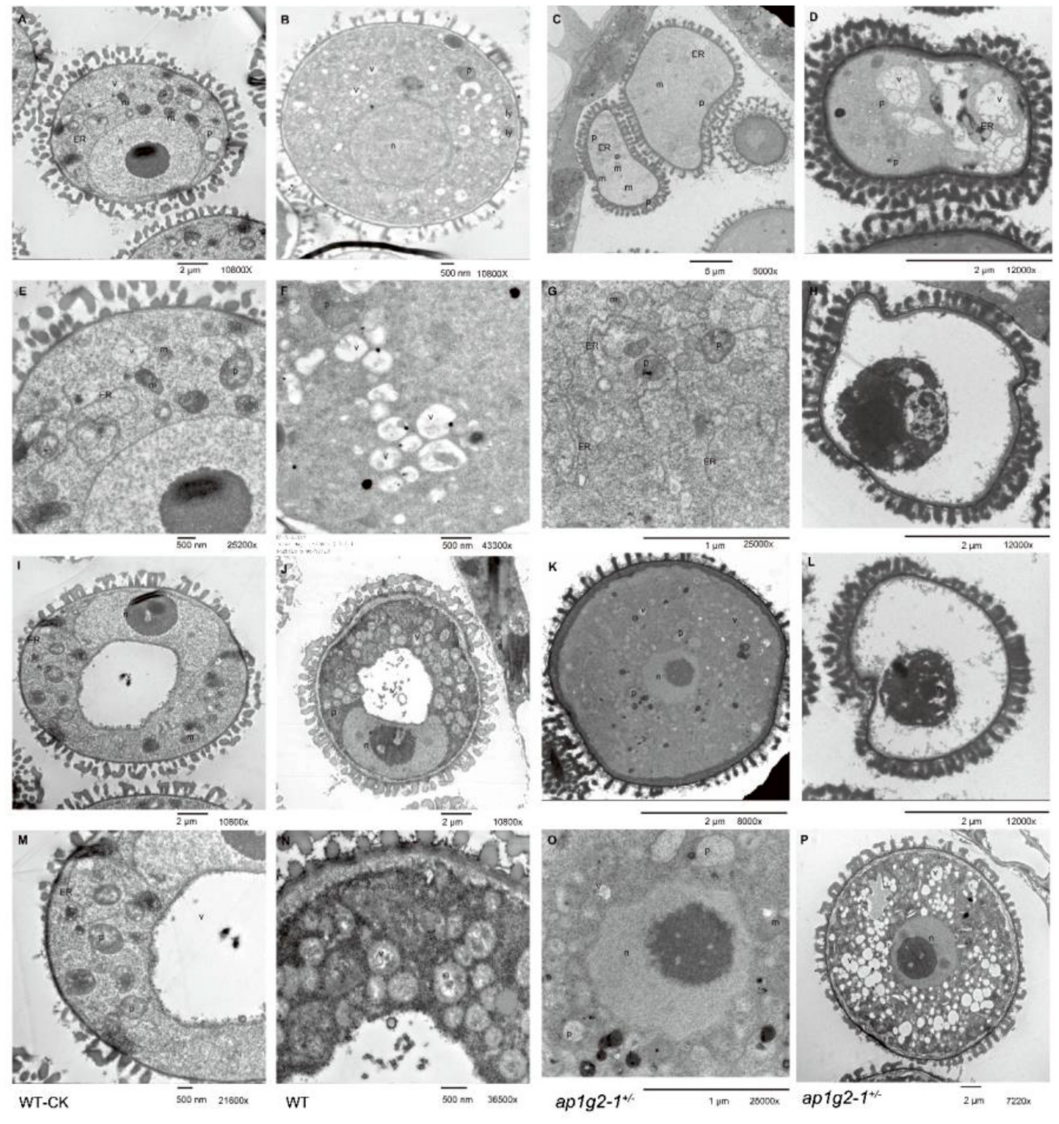
TEM of pyroantimonate-labeled microspores from the wild type and *ap1g2-1*^+/−^. **(A,I)** The microspores before pollen mitosis I from the wild type without potassium pyroantimonate treatment. **(E,M)** The magnifications of **(A,I)**. **(B,F,J)** The microspores before PMI from the wild type with potassium pyroantimonate treatment. **(N)** The magnifications of **(J)**. **(C,D,G,H,L)** Abnormal microspores failed to undergo pollen mitosis I from *ap1g2-1*^+/−^. **(K,O,P)** were normal biocellular pollens from *ap1g2-1*^+/−^. **(G,O)** were the magnifications of **(C,K)**. n, nucleus; ER, endoplasmic reticulum; p, proplastids; v, vacuoles; m, mitochondria; ap, autophagosomes; a, amyloplasts; ly, lysosome.

To investigate the distribution of calcium precipitates in the abnormal developing microspores of *ap1g2*, anthers at different stages from the wild type and *ap1g2-1*^+/−^ were treated with potassium pyroantimonate with non-treated wild-type anthers as control. Through TEM, very few black dots were observed in the control wild-type pollen grains (Figures 11A,E,I,M, Supplementary Figure 4B). A number of calcium precipitates appeared in the cytoplasm of well-developing microspores from the wild type (Figures 11B,F,J,N, Supplementary Figure 4A) and *ap1g2-1*^+/−^ (Figures 11K,O,P), while, in the aborted microspores of *ap1g2-1*^+/−^, we observed few calcium precipitates (Figures 11C,D,G, Supplementary Figure 4A).

The ultrastructures of the microspores from the wild type and *ap*1*g*2−1^+/−^ also confirmed that the infertile pollen grains were arrested before PMI (pollen mitosis I). After its microspores were released from tetrad, about half the pollen grains had abnormal shapes and failed to form a large vacuole (Figure 11D). In these sterile microspores, there was numerous endoplasmic reticulum (ER) distributed in the cytoplasm (Figure 11G), and then some abnormal vacuoles occurred inside the circles formed by ER (Figure 11D). The abnormal vacuolization led to the degradation of the cytoplasm compartments (Figures 11H,L).

## Discussion

In angiosperm, the gametophytes consist of fewer cells and are embedded within the sexual organs. The female gametophyte develops inside the ovule, while the male gametophyte, also called the pollen, develops in the anthers. Out of the −26,000 genes recognized in Arabidopsis, only a few thousand have been functionally defined (Bouche and Bouchez, 2001), while more than 30% of the expected Arabidopsis genes have not been studied with confidence. Earlier, various studies have been conducted related to gametophyte development. Here, we report the *AP1G2* gene and its essential role in controlling female and male gametophyte development.

*AP1G2* encodes one of the largest subunits of a heterotetrameric protein complex, which sorts proteins at the trans-Golgi network and endosomes (Park et al., 2013). The *AP1G* subunit is encoded by two homologs, *AP1G1* and *AP1G2*, which are both constitutively expressed in male and female reproductive cells (Wang et al., 2017). We obtained two lines, *ap1g2-1*^+/−^ and *ap1g2-3*^−/−^, which showed partial functional megaspore and microspores failed to undergo the first mitosis. A single T-DNA insertion in exon 7 of *ap1g2-1*^+/−^ showed a nearly 50% seed set and no homozygous plants obtained in the self-pollinated progeny. While *ap1g2-3* mutant with insertion in 3′ UTR (56 bp upstream from the poly A tail of mRNA), only the homozygous plants showed defective phenotypes in female and male gametophyte development, which were more severe than those of *ap1g2-1*^+/−^. The defects of *ap1g2-3* were relatively weak compared with the *ap1g2-1* mutant line, which was likely due to its rearward mutation position or other insertion mutations. The reason of caused-impaired function of AP1G2 might be that some miRNAs can decrease gene expression of mRNAs by binding to specific sites within the 3′ UTR, which leads to either inhibiting translation or directly causing degradation of the transcript (Barrett et al., 2012; Pichon et al., 2012). In this work, we found that insertion mutation in 3′ UTR of *AP1G2* decreased its expression level in various organs. This result supports that the regulatory regions within the 3′ UTR could influence translation efficiency of the mRNA. The EMS mutant had additional integument defects, which were more likely caused by other mutations, which downregulated *INO*, the key regulatory gene of the outer integument because the two T-DNA mutants did not show the phynotype and decrease of *INO* expression. Moreover, the transgenic plants of *ap1g2-4* carrying wild-type *AP1G2* driven by the native promoter still lacked the outer integuments.

Female gametophyte mutations fall into two classes, including the female gametophyte-specific class and the general female gametophytic class (Drews et al., 1998). *ap1g2* mutants belong to the second class, including male gametophyte developmental defects too. The ultrastructure of female gametophytes and male gametophytes from the wild type and *ap1g2-1*^+/−^ showed the failure of some fundamental cellular processes of mutant gametophytes, such as mitosis, vacuole formation, cell expansion, and subcellular migration. *ap1g2*-impaired microspores were found without the formation of a large vacuole and an asymmetric cell division, but performing abnormal vacuolization, vacuole collapse, and cytoplasmic degradation. In the *ap1g2*-defect embryo sacs, neither the first mitosis nor the formation of a big vacuole that characterizes the FG3 stage was observed. These results suggested that *ap1g2* mutations were responsible for the failure of mitosis and the normal large vacuole formation in both female and male gametophytes. The phenotypes of *ap1g2* had a resemblance with those of the insertion lines in *VACUOLELESS GAMETOPHYTES* (*VLG*) as a DC1 domain-containing protein present in the endomembrane system (D'ippolito et al., 2017). VLG localized to plant prevacuolar compartments (PVCs) or multivesicular bodies (MVBs), which mediated protein trafficking to vacuoles in the secretory pathway and were also considered late endosomes in the endocytic pathways. The cytosolic adaptor protein-1 complex (AP-1) that was found localized on the trans-Golgi network (TGN)/endosomal membranes also played an essential role in protein trafficking between the TGN and endosomes by specific sorting signals (Bonnemaison et al., 2014; Wang et al., 2014, 2017). They suggested that the post-Golgi traffic pathway is crucial for gametophyte development.

Transcriptome analysis was carried out using the ovules at various stages from the wild type, *ap1g2-1*^+/−^, *ap1g2-3*^−/−^, and heterogenous homozygous mutant *ap1g2-1*^+/−^*/ap1g2-3*^+/−^. We used STRING analysis to predict the associations of the proteins of DEGs (Szklarczyk et al., 2019), which showed AP1G2 was predicted to have direct interaction with PICALM5A/B and PICALM9A/B, which were members of the PICALM (phosphatidylinositol binding clathrin assembly protein) subfamily. PICALM, is a clathrin adaptor protein containing functions in clathrin-coated vesicle formation (Ford et al., 2001; Meyerholz et al., 2005; Chen et al., 2011; De Craene et al., 2012; Muro et al., 2018). The PICALM subfamily has 18 group members (Muro et al., 2018), of which the members showed different expression patterns during the development of ovules in the wild type. *PICLAM4A, PICLAM3*, and *PICLAM1A/B* maintained consistent expression, indicating that they may provide basic and consistent functions, whereas members, including *PICALM5A/B* and *PICALM9A/B*, together with *PICALM10C*, seemed to be involved in the megagametogenesis and pollination because they showed a low expression level before the FG1 stage. However, they also maintained relatively higher transcriptional activity during the mitosis process of the female gametophyte and peaked when the matured female gametophytes were ready for the double fertilization. While the others showed extremely low transcriptional activity throughout the ovule development. These data demonstrated that PICALM proteins played roles during development by job division and coordination, and several members might act redundantly. It has been reported that *via* clathrin-mediated endocytosis, PICALM5A/B could recycle ANXUR kinases for the pollen tube integrity (Muro et al., 2018), and PICALM9B (EAP1) could antagonize REN4 by directly targeting and removing plasma membrane-localized REN4 (ROP1 Enhancer 4, AT2G26490) in pollen tubes (Li et al., 2018). The protein-protein interaction assays both *in vitro* and *in vivo* determined the four PICALM proteins directly interacted with AP1G2. Moreover, AP1G2 and the four PICALM proteins were found co-localized on the membrane. Thus, the complex formed with AP1G2 and four PICALM proteins might be required for gametophyte development *via* clathrin-mediated endocytosis.

AP complexes exhibit highly conserved roles in vesicular transport and act as a major hub of interactions in many organisms (Teh et al., 2013). There are five AP complexes (AP1–AP5) identified with conserved four heterotetramers structural compositions in the Arabidopsis genome (Teh et al., 2013; Wang et al., 2014). Reports for the AP-1 complex indicated that AP-1 is essential for viability in multicellular organisms. In mice, homozygous disruptions of the genes encoding γ1 or μ1A caused embryonic lethality (Zizioli et al., 1999; Meyer et al., 2000). In yeast, mutant *cis1-1/apm1-1*, an allele of the mammalian μ1A subunit of the AP-1 complex, was synthetically lethal, with a deletion of calcineurin, leading to pleiotropic defects in cellular processes, such as secretion, cytokinesis, vacuolar fusion, and cell wall integrity (Kita et al., 2004). In Arabidopsis, AP-1 has two putative copies of each adaptinβ (At4g11380 and At4g23460), γ (At1g60070 and At1g23900), μ (At1g60780 and At1g10730), and σ (At2g17380 and At1g35410). The medium subunit of AP1, redundant AP-1 μ-adaptins AP1M1 and AP1M2, were reported complexed with large subunits γ-adaptin of the heterotetrameric AP-1. The knockout mutation *ap1m2* displayed impairing pollen function and arrested plant growth, and the *ap1m1ap1m2* double mutant was nearly pollen-lethal (Park et al., 2013). Analysis of a double knockout *ap1g1 g2/*+ indicates *AP1G* was important to synergid-controlled pollen tube reception and pollen development by mediating vacuolar remodeling (Feng et al., 2017; Wang et al., 2017). Many DEGs of the three *ap1g2* ovules were enriched on pollination, indicating AP1G2's role in pollen tube reception. And our study determined that the *ap1g2* mutant had sterility defects in both female and male gametophyte development. Previous studies have indicated that AP-1 is required to execute somatic cytokinesis properly in root and shoot cells in Arabidopsis (Teh et al., 2013). *AP1M1* promotes secretory and vacuolar trafficking that is required for cell division and growth during both pollen development and plant growth (Park et al., 2013). The expression of *AP1G2* was also detected in the tissues of active cell division, for example, shoot apical meristems, and leaf primordial and root tips. Abnormal vacuolation occurred in both aborted female and male gametophytes, which failed to undergo mitosis. Therefore, we speculated that AP1G2 might have multiple functions and be involved in regulating the cell division cycle.

Calcium ion (Ca^2+^) signaling is essential for cells and is used for the crucial process by shifting a low concentration of Ca^2+^ to an increased level to activate or change some proteins that trigger downstream events. The importance of Ca^2+^ signaling in sexual reproduction has been widely reported, especially in pollen germination, guidance, and pollen tube growth (Iwano et al., 2012; Mahs et al., 2013; Steinhorst and Kudla, 2013; Lenartowski et al., 2015; Steinhorst et al., 2015; Wang et al., 2015; Zheng et al., 2019). Cytosolic calcium transient also occurred in the egg and central cells of female gametophytes to trigger pollen tube burst and ensure successful fertilization (Denninger et al., 2014; Hamamura et al., 2014). The changes in cytosolic free Ca^2+^ were detected during the processes of megasporogenesis and megaspore degeneration in lettuce (Qiu et al., 2005), but little is known about Ca^2+^ dynamics during megagametogenesis. In this study, we showed that the Ca^2+^ concentration was higher in normal developing female and male gametophytes than that in *ap1g2*-defective gametophytes, which revealed that free Ca^2+^ signaling was crucial for the process of gametogenesis. A genetic screen was carried out for mutations that were synthetically lethal, with a deletion of calcineurin that is a regulator of Ca^2+^ signaling in fission yeast, and *apm1-1*, an allele of the mammalian μ1A subunit of the AP-1 complex, was identified (Kita et al., 2004). Our transcriptomic analysis revealed that some *RALFL* genes, which lead to calcium-dependent signaling events through the transient increase of the cytoplasmic Ca^2+^ concentration, and *CML16*, a member of the calcium-binding EF-hand protein family, together with several calcium-dependent protein kinases, including *CPK14, CPK16*, and *CPK17*, were all downregulated in the *ap1g2-1*^+/−^ mutant. And it has been reported that endocytosis is a calcium-dependent process. The Ca^2+^ channel could regulate clathrin-mediated endocytosis (Yao et al., 2017). Intracellular calcium may also play a role in the function of clathrin-coated vesicles (Nathke et al., 1990). Therefore, the Ca^2+^ dynamic might affect the clathrin-mediated endocytosis in the process of gametogenesis.

## Data Availability Statement

The clean data has been uploaded in SRA. The BioProjected ID: PRJNA849024: Arabidopsis ovule RNAseq data.

## Author Contributions

RM and YZ designed the research. YZ, WF, and HC conducted the research. YZ, ZP, and N-U-A analyzed the data. YZ wrote the manuscript. RM, L-YC, and M-CC guided the experiment and revised the manuscript. All authors approved the final version of the manuscript for publication.

## Funding

This work was supported by National Science Foundation (NSF) Plant Genome Research Program Award DBI-1546890 to RM.

## Conflict of Interest

The authors declare that the research was conducted in the absence of any commercial or financial relationships that could be construed as a potential conflict of interest.

## Publisher's Note

All claims expressed in this article are solely those of the authors and do not necessarily represent those of their affiliated organizations, or those of the publisher, the editors and the reviewers. Any product that may be evaluated in this article, or claim that may be made by its manufacturer, is not guaranteed or endorsed by the publisher.
