# Supplementary material for: AP1G2 Affects Mitotic Cycles of Female and Male Gametophytes in Arabidopsis
Source: Front Plant Sci. 2022 Jul 7;13:924417. doi: 10.3389/fpls.2022.924417 (PMC9301471; doi:10.3389/fpls.2022.924417)

**Table. S1.** The pimers for identification of candidate causal SNPs

| Number | Potential SNPs | Primer name | Primer sequence | Production size |
| --- | --- | --- | --- | --- |
| 1 | 3038354 | At1g09415F | ACATATTTCCGCATITCCC | 422bp |
|  |  | At1g09415R | GCTGTGCCATTCCCTGT |  |
| 2 | 3617508 | At1g10870F | TTTGTGGTTCCCTGAT | 378bp |
|  |  | At1g10870R | CTTTCTGCTGCGAGTA |  |
| 3 | 3921980 | At1g11660F | TGATGGAGGAGAAGGAC | 378bp |
|  |  | At1g11660R | GCGAAAGGGTAAGAAT |  |
| 4 | 4237949 | At1g12430F | AAACACCCTATACCAAA | 484bp |
|  |  | At1g12430R | AGATTAAGGCCACGT |  |
| 5 | 4251504 | At1g12470F | ATCAAGGAAGGCAAGT | 385bp |
|  |  | At1g12470R | CACGGCATTAACGAG |  |
| 6 | 4425357 | At1g12970F | AGGGAGACTCACAAACC | 462bp |
|  |  | At1g12970R | AGCTGCACCACCATG |  |
| 7 | 5393968 | At1g15680F | GCTTGGTGGATAAACATAA | 353bp |
|  |  | At1g15680R | GCTACTCCGACAACGATA |  |
| 8 | 5409843 | At1g15730F | TAGCCTCACCTTACCAG | 404bp |
|  |  | At1g15730R | GTCCGAACCTTCACAG |  |
| 9 | 5606176 | At1g16400F | CCGATGGTTAGGTGGTTG | 362bp |
|  |  | At1g16400R | CGATCGCTGCTATACAAA |  |
| 10 | 5969995 | At1g17140F | GATTCGGGTTTTAGCCCTTTT | 425bp |
|  |  | At1g17140R | TTGCTCCATAACCTAAACAC |  |
| 11 | 6152609 | At1g17880F | GATTCTGGTCGGAAACTA | 246bp |
|  |  | At1g17880R | GAAACAGGCTTCTGGTGA |  |
| 12 | 7912351 | At1g22410F | ACCACCGTCAACCAAA | 344bp |
|  |  | At1g22410R | CAGAAACGCTTCACCC |  |
| 13 | 8047912 | At1g22730F | AAAGGGTTTAGCAGAATC | 405bp |
|  |  | At1g22730R | TCTGACATCTTTGGGAGG |  |
| 14 | 8444076 | At1g23900F | TCTAACCGCGACAATAAC | 465bp |
|  |  | At1g23900R | GAAGCATCTGGGTCCTAT |  |
| 15 | 9393820 | At1g27050F | CGAGTATGAGCCGCAAGA | 336bp |
|  |  | At1g27050R | CGATGGAGATACCGAAAG |  |
| 16 | 9506838 | At1g27370F | AATCTATGTTGCTGGAAG | 478bp |
|  |  | At1g27370R | GTATCTGTTTGGGTTGC |  |
| 17 | 9542379 | At1g27470F | TCAGTGCTACGGTTTCTC | 432bp |
|  |  | At1g27470R | GTTGTTTGGCTGTTATTGT |  |
| 18 | 10198968 | At1g29179F | ACTTCGTAGTCGGACAG | 476bp |
|  |  | At1g29179R | GTGAATTAGGCTTTGG |  |
| 19 | 10430110 | At1g29790F | GTGTCACTACCTCCAAGAT | 479bp |
|  |  | At1g29790R | ATGAAGAAGGTCAGGGT |  |
| 20 | 10716662 | At1g30360F | TTGATTTGGGTTTGAG | 468bp |
|  |  | At1g30360R | ATAGGCAGAATGGAGTT |  |
| 21 | 11339217 | At1g31670F | CTCGTCCGTAACATCAT | 449bp |
|  |  | At1g31670R | TTCACCGACCCATTC |  |
| 22 | 11564394 | At1g32140F | GGCTCTATCTTAGTTGTCA | 465bp |
|  |  | At1g32140R | AGTTTGGAACCCGTAT |  |
| 23 | 12450362 | At1g34180F | CCTCTGGTATGGCACCTCTT | 552bp |
|  |  | At1g34180R | AAACTAATGCTTCGTGGG |  |
| 24 | 13055969 | At1g35480F | AAGCTAAACCGCCAAATC | 490bp |
|  |  | At1g35480R | GGCTCTAAGTATCCCACA |  |
| 25 | 15863731 | At1g42396F | ACACCGTCCTCGGAAACTAA | 439bp |
|  |  | At1g42396R | GCCACAACACTCCACCCTCT |  |
| 26 | 15896290 | At1g42440F | GAAGCCAACGAGAAACAC | 433bp |
|  |  | At1g42440R | TGCTGCCTCTAAACTATC |  |
| 27 | 17100238 | At1g45180F | AGCCCAGCAATGACAGCA | 450bp |
|  |  | At1g45180R | CTGGGACAAACAAGGGAAT |  |
| 28 | 17141223 | At1g45223F | ATCGTATTCAAGTCCCATTA | 752bp |
|  |  | At1g45223R | GTCGTGGCAACAAGACTCAA |  |
| 29 | 18779760 | At1g50690F | CCGTGATGGGCAAATAAGAC | 535bp |
|  |  | At1g50690R | TCGACTCGCTGTACGAAGAC |  |
| 30 | 25046783 | At1g67080F | AGATGAAGACAAAGGCTGAG | 253bp |
|  |  | At1g67080R | ATAAAGATGGAAGGATGAGC |  |

Table S2. The genomic sequence-specific primers of T-DNA insertion lines

| Candidate genes | T-DAN lines | Insertion position | The genomic sequence-specific primers |
| --- | --- | --- | --- |
| At1g22410 | SALK_026183 | 2nd exon | LP CCAGGCTTATTGTCAGCATTC |
|  |  |  | RP AGGATTCCTGATCCACAGAGG |
|  | SALK_028368 | Last exon | LP TCCTTGTAGGACGACTCTTCG |
|  |  |  | RP CTAGCAAAGCAAAATCGGTTG |
|  | SALK_127811 | Promoter | LP TTACGACGGGTGTTAGGTGAG |
|  |  |  | RP ATGAAGCAGCTGGTGTITTTG |
| A11g22730 | SALK_025678 | Last exon | LP AGACACCGAATTGAATGCATC |
|  |  |  | RP CGTCATCAAAAAGTATCCAAAAC |
|  | SALK_022908 | Last exon | LP AGACACCGAATTGAATGCATC |
|  |  |  | RP TTTGTCCAGCTTITAAGAGAGG |
|  | SALK_152280 | Last exon | LP AGACACCGAATTGAATGCATC |
|  |  |  | RP GGGGTITGTCCAGCTTITAAG |
| At1g23900 | SALK_032500 | 7th exon | LP GAGCTCAAGAAGCAACAATCC |
|  |  |  | RP ACTTTGTTATCCTGGTTCTITG |
|  | SALK_032502 | 7th exon | LP GAGCTCAAGAAGCAACAATCC |
|  |  |  | RP ACTTTGTTATCCTGGTTCTTTG |
|  | SALK_137129 | 3'UTR | LP GAGTCACTAACAGCCAGCAGG |
|  |  |  | RP TTATGAATTTCGCATCAAGCC |

Table S3 Synchrony of female gametophytes in *ap1g2-3^-/-^* and *ap1g2-4^-/-^* pistils.

| Pistil number | No. of female gametophytes at developmental stages in *ap1g2-3^-/-^* pistils | | | | | | | | |  | | No. of female gametophytes at developmental stages in *ap1g2-4^-/-^* pistils | | | | | | | |  |
| --- | --- | --- | --- | --- | --- | --- | --- | --- | --- | --- | --- | --- | --- | --- | --- | --- | --- | --- | --- | --- |
|  | MMC | FG1 | FG2 | FG3 | FG4 | FG5 | FG6 | FG7 | No nuclei | Total FGs | MMC | FG1 | FG2 | FG3 | FG4 | FG5 | FG6 | FG7 | No nuclei | Total FGs |
| P1 | 41 |  |  |  |  |  |  |  |  | 41 | 50 |  |  |  |  |  |  |  |  | 50 |
| P2 | 18 | 28 |  |  |  |  |  |  |  | 46 | 40 | 11 |  |  |  |  |  |  |  | 51 |
| P3 | 1 | 36 | 1 |  |  |  |  |  | 5 | 43 | 6 | 37 | 2 |  |  |  |  |  |  | 45 |
| P4 | 1 | 35 | 1 |  |  |  |  |  | 9 | 46 | 10 | 35 | 1 |  |  |  |  |  |  | 46 |
| P5 | 1 | 45 | 1 |  |  |  |  |  | 9 | 56 |  | 28 | 3 | 7 |  |  |  |  | 6 | 44 |
| P6 |  | 29 | 3 |  |  |  |  |  | 3 | 35 |  | 7 | 2 | 7 | 10 |  |  |  | 19 | 45 |
| P7 |  | 30 | 2 | 4 |  |  |  |  | 7 | 43 |  | 7 | 4 | 11 | 10 | 1 |  |  | 18 | 51 |
| P8 |  | 32 | 2 | 2 | 4 |  |  |  | 9 | 49 |  | 2 |  | 6 | 8 | 7 | 4 |  | 20 | 47 |
| P9 |  | 16 | 1 | 1 | 3 | 3 | 1 | 1 | 10 | 36 |  | 7 |  | 5 | 11 | 3 | 4 | 3 | 12 | 45 |
| P10 |  | 27 |  | 1 | 3 | 1 | 1 | 2 | 19 | 54 |  | 3 |  | 7 | 7 | 2 | 2 | 13 | 13 | 47 |
| P11 |  | 21 | 1 |  | 1 | 2 |  | 2 | 16 | 43 |  | 7 |  | 1 | 1 |  | 2 | 17 | 27 | 55 |
| P12 |  | 21 |  |  | 1 | 2 | 5 | 5 | 15 | 49 |  | 6 |  | 2 |  | 1 | 4 | 9 | 25 | 47 |
| P13 |  | 16 |  |  |  | 1 | 1 | 12 | 20 | 50 |  | 5 |  |  |  | 1 | 2 | 15 | 21 | 45 |
| P14 |  | 15 |  |  |  |  | 1 | 10 | 23 | 49 |  | 6 |  |  |  |  |  | 19 | 26 | 51 |

**Fig S1.** Pollen development was impaired in *apg2-3^-/-^* mutants. (A, B) Scanning electron micrographs (SEMs) of anthers from WT and *ap1g2-3^-/-^*, bars = 200 μm. (C, D) SEMs of pollen grains from WT and *ap1g2-3^-/-^*, bars = 30 μm. (E, F) Pollen grains germination in vitro of WT and *ap1g2-3^-/-^*, respectively, bars = 100 μm. Arrows point at aborted pollen grains.
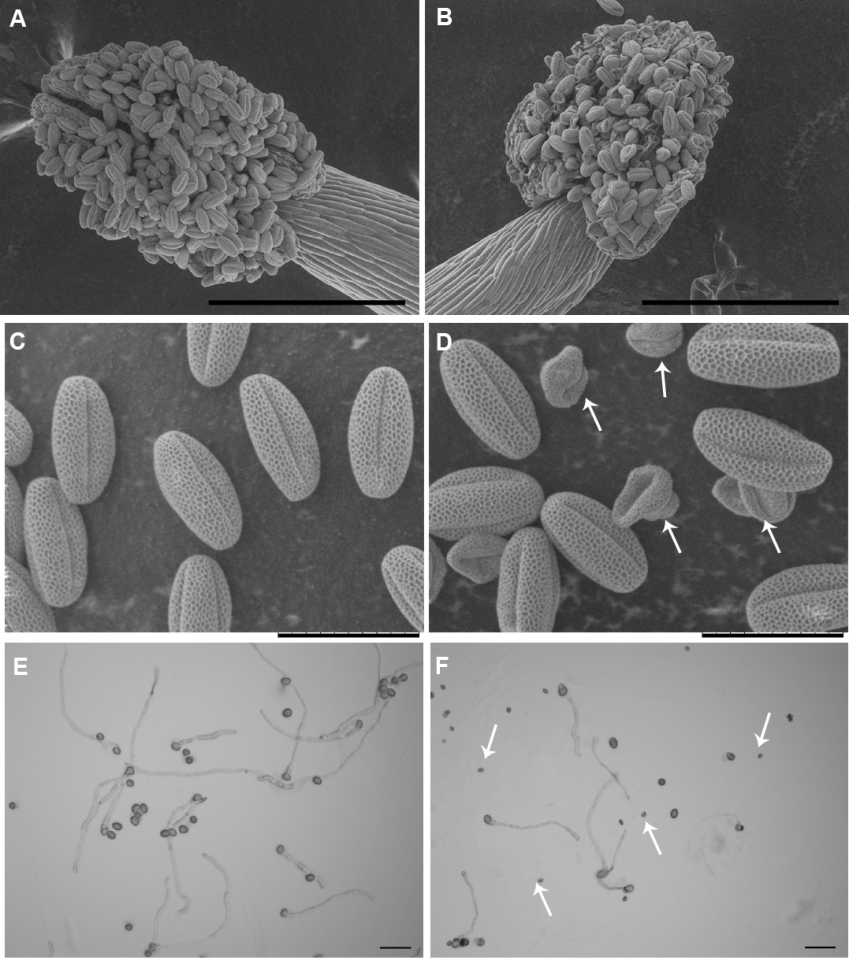


**Fig S2** (A) Principal component analysis (PCA) plot allowing visualization of the differences in the expression profiles between samples. PCA derived from the FPKM of all genes. PC1, principal component 1; PC2, principal component 2. Explained variants PC1: 28.74%, PC2: 18.53%. R^2^ values > 0.75 are generally interpreted as clearly separated, R^2^ > 0.5 as separated and R < 0.25 as groups hardly separated. (B) Differentially expressed genes at each stage of *ap1g2-1^+/-^*, *ap1g2-1^+/-^/ap1g2-3^+/-^* and *ap1g2-3^-/-^* ovule development. Red: up-regulated DEGs; green: down-regulated; blue: total DEGs. (C) Venn diagram of total DEGs of various stages between three mutants. *ap1g2-1^+/-^*, *ap1g2-1^+/-^/ap1g2-3^+/-^* and *ap1g2-3^-/-^* were represented by M1, M2 and M3, respectively.


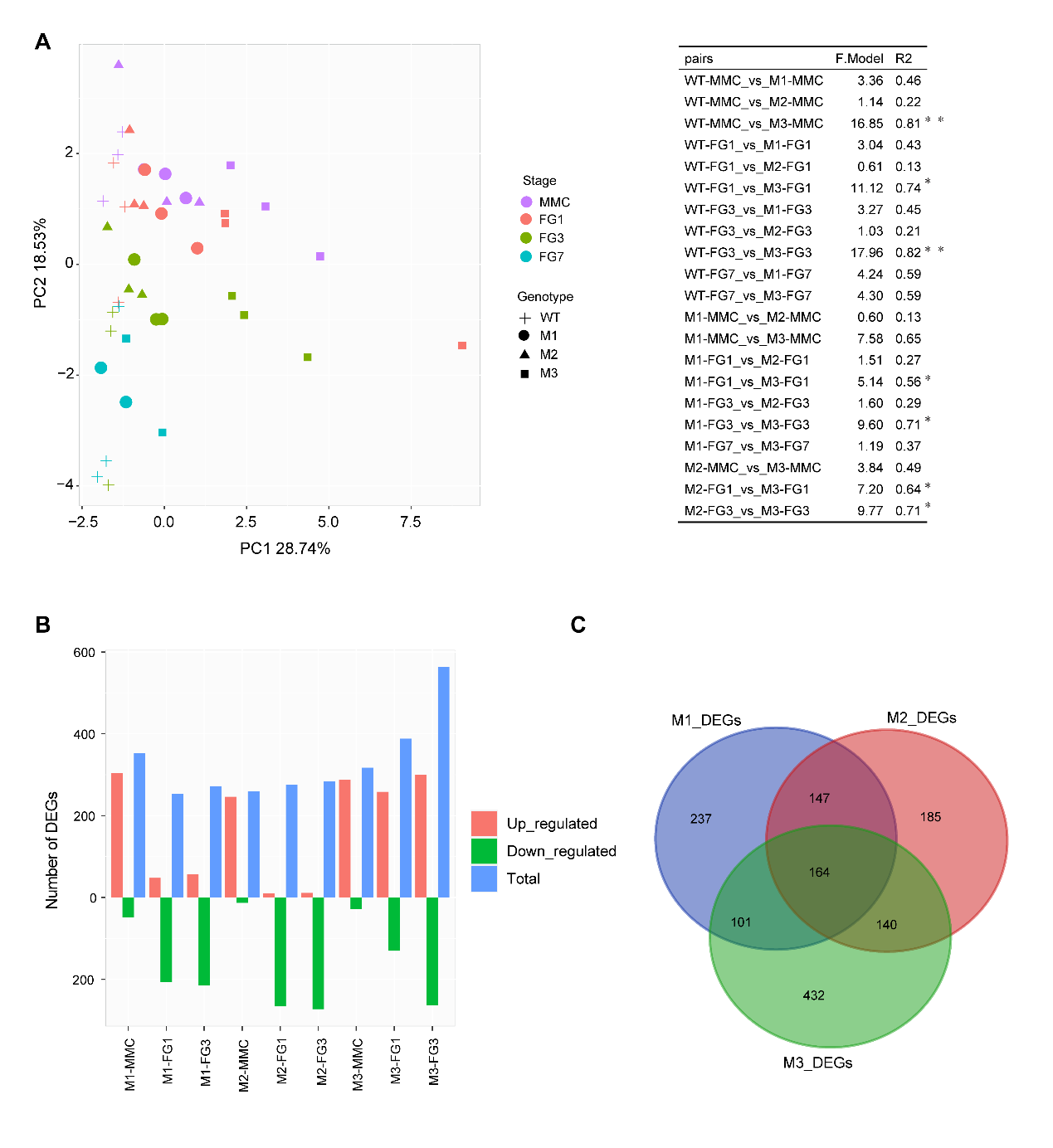


**Fig. S3.** Fuzzy c-means clustering of expression profiles. The x axis represented three developmental stages of the wild-type, *ap1g2-1^+/-^*, *ap1g2-1^+/-^/ap1g2-3^+/-^* and *ap1g2-3^-/-^*, and the y axis represents log2-transformed, normalized intensity ratios in each stage.


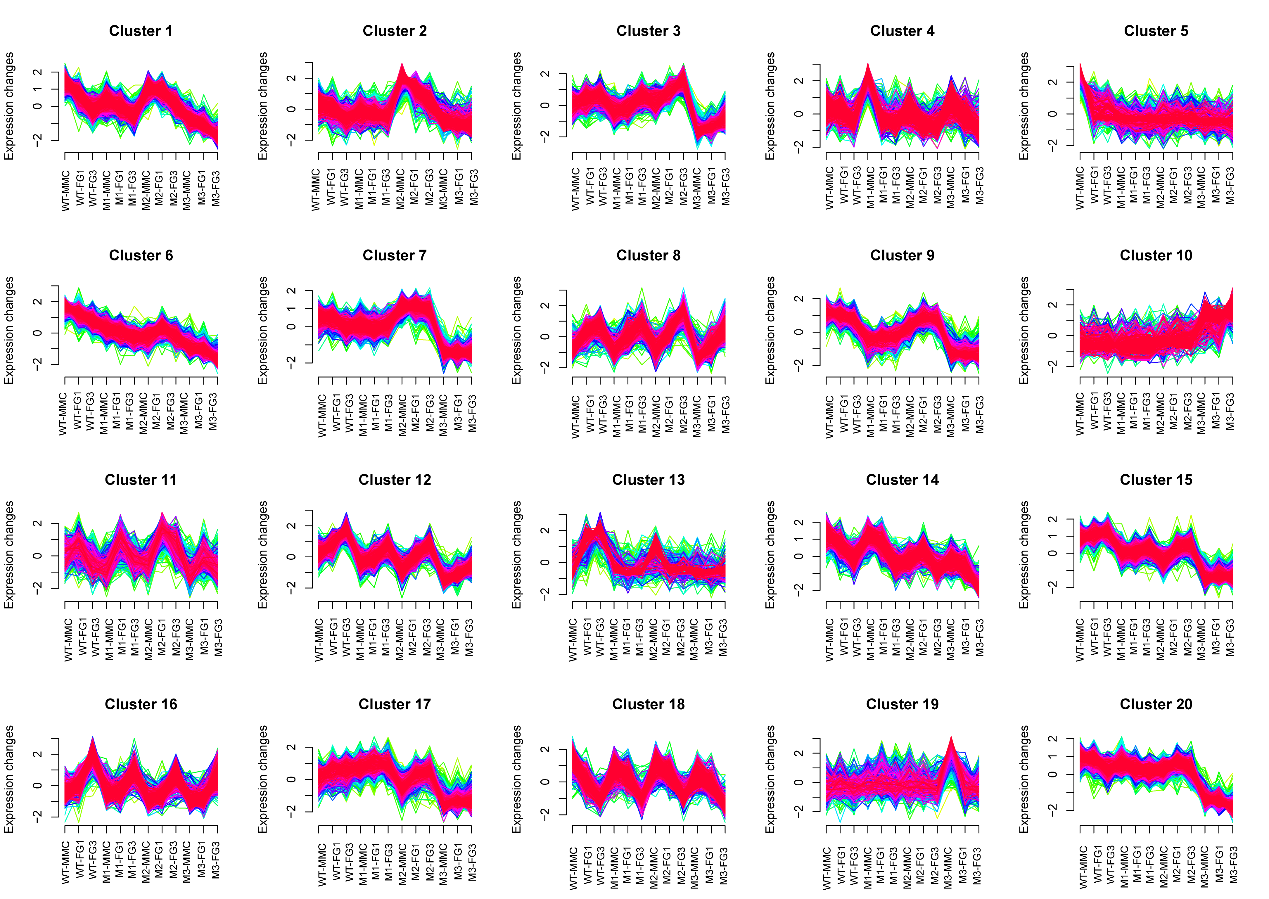


**Fig. S4 .** Quantitative real time PCR validation of *AP1G2*, PICLAM5A/B and PICLAM9A/B.

**
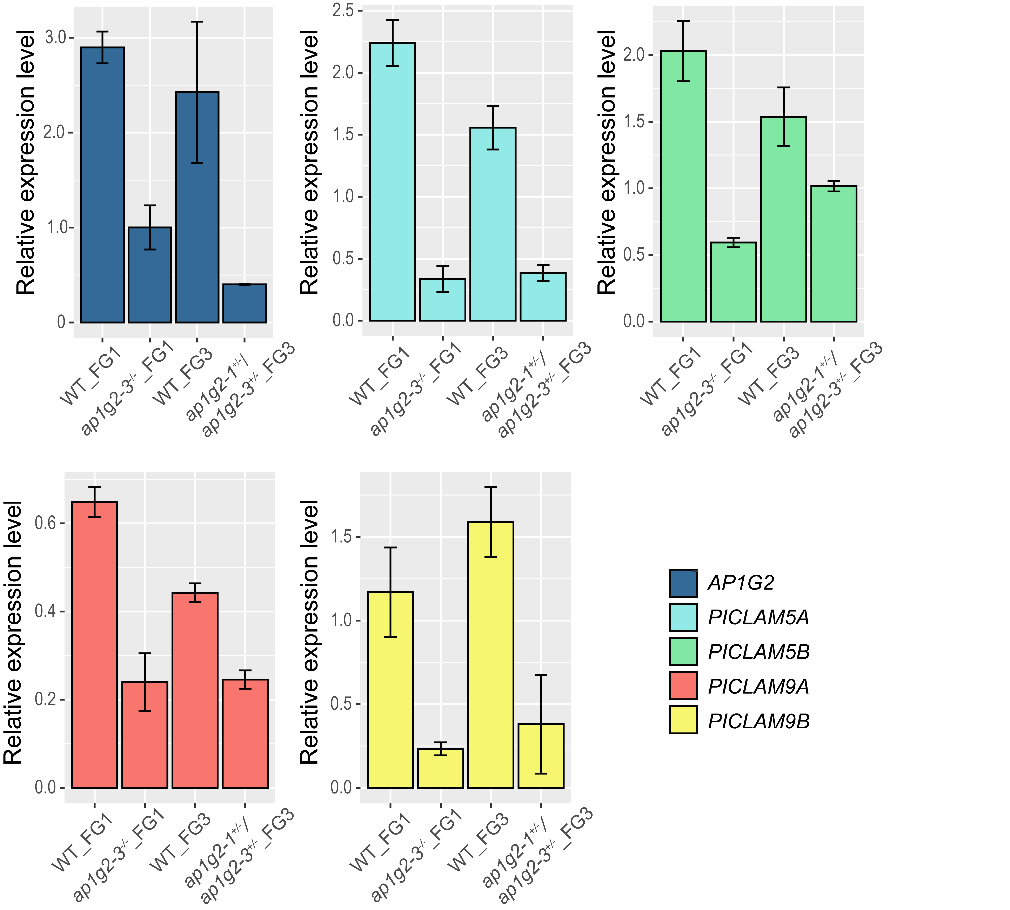
**

**Fig. S5 .** Relative abundance of calcium precipitates in the normal and aborted embryo sacs and microspores from WT and *ap1g2-1^+/-^* respectively. Each result was the average of at least three sectional profiles. Error bars indicate standard error. * means significant different (P<0.05); ** means very significant different (P<0.01);*** means very significant different (P<0.001).WT-CK in (B) means the not pyroantimonate-labeled wild-type microspore.


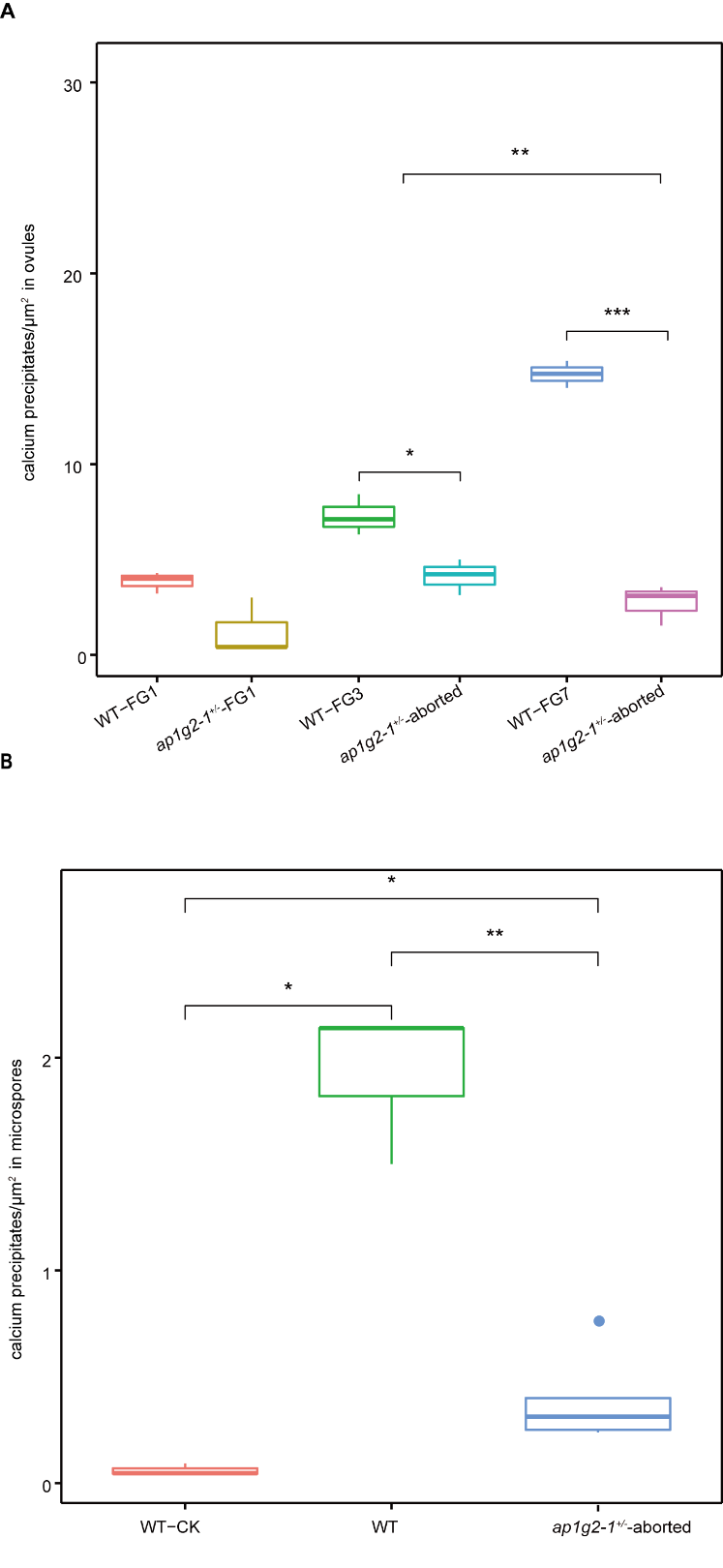

Supplement: Supplementary file 1 [file Data_Sheet_1.docx]
